# Periodontitis aggravates COPD through the activation of γδ T cell and M2 macrophage

**DOI:** 10.1128/msystems.00572-23

**Published:** 2024-01-12

**Authors:** Kaixin Xiong, Keping Ao, Wei Wei, Jiajia Dong, Jia Li, Yutao Yang, Boyu Tang, Yan Li

**Affiliations:** 1 State Key Laboratory of Oral Diseases & National Center for Stomatology & National Clinical Center for Oral Diseases, West China Hospital of Stomatology, Sichuan University, Chengdu, China; 2 Department of Laboratory Medicine, West China Hospital, Sichuan University, Chengdu, China; 3 Department of Prosthodontics, Beijing Stomatological Hospital, School of Stomatology, Capital Medical University, Beijing, China; 4 Department of Pulmonary and Critical Care Medicine, West China Hospital, Sichuan University, Chengdu, China; 5 Department of Conservation Dentistry and Endodontics, West China Hospital of Stomatology, Sichuan University, Chengdu, China; NIAID, NIH, Bethesda, Maryland, USA

**Keywords:** γδ T, M2 macrophage, periodontitis, COPD, IL 17, IFN γ

## Abstract

Chronic obstructive pulmonary disease (COPD) is a chronic systemic inflammatory disease with high morbidity and mortality. Periodontitis exacerbates COPD progression; however, the immune mechanisms by which periodontitis affects COPD remain unclear. Here, by constructing periodontitis and COPD mouse models, we demonstrated that periodontitis and COPD could mutually aggravate disease progression. For the first time, we found that the progression was associated with the activation of γδ T cells and M2 macrophages, and M2 polarization of macrophages was affected by γδ T cells activation. In the lung tissues of COPD with periodontitis, the activation of γδ T cells finally led to the increase of IL 17 and IFN γ expression and M2 macrophage polarization. Furthermore, we found that the periodontitis-associated bacteria *Porphyromonas gingivalis* (*P. gingivalis*) promoted the activation of γδ T cells and M2 macrophages *ex vivo*. The data from clinical bronchoalveolar lavage fluid (BALF) samples were consistent with the *in vivo* and *ex vivo* experiments. For the first time, our results identified the crucial role of γδ T-M2 immune mechanism in mediating periodontitis-promoted COPD progression. Therefore, targeting at periodontitis treatment and the γδ T-M2 immune mechanism might provide a new practical strategy for COPD prevention or control.

**IMPORTANCE:**

Periodontitis exacerbates chronic obstructive pulmonary disease (COPD) progression. For the first time, the current study identified that the impact of periodontitis on COPD progression was associated with the activation of γδ T cells and M2 macrophages and that M2 polarization of macrophages was affected by γδ T cells activation. The results indicated that targeting at periodontitis treatment and the γδ T-M2 immune mechanism might provide a new practical strategy for COPD prevention or control.

## INTRODUCTION

Chronic obstructive pulmonary disease (COPD) is a chronic systemic inflammatory disease characterized by airflow obstruction, pulmonary function deterioration, cough with sputum, and shortness of breath, which is progressive and not completely reversible (1, 2). It is a major cause of morbidity and disability worldwide and is considered the third leading cause of death globally (3, 4). Periodontitis is a chronic infectious disease caused by dental plaque biofilm and acts as a potential risk factor for a variety of systemic diseases, such as cardiovascular disease, diabetes, hypertension, various cancers, and chronic obstructive pulmonary disease (5–9). Periodontitis is a microbial biofilm-associated infectious disease. *Porphyromonas gingivalis (P. gingivalis*) is related to the severity of periodontal disease and has been identified as one of the critical virulence factors of periodontitis (10). Even at low abundance, *P. gingivalis* can induce chronic periodontitis through interference with host immune responses and causing dysbiosis of the oral commensal bacterial community (10–12), and recently, it has been identified as the keystone bacteria in the dysbiosis of oral microbiota and periodontitis occurrence and development (10–12).

Oral health status seriously impacts respiratory diseases via several aspects (13–15): (i) Changes in oral health status leads to oral immune imbalance, after which local oral immune cells migrate to the lung, causing an immune imbalance of lung tissue and, thus, affect lung diseases; (ii) Changes in oral health status can also promote the progression of lung disease by affecting systemic inflammation. (iii) Oral health status is closely related to changes of oral microbiota, and then oral microorganisms affect the lung flora through inhalation and colonization on lung tissue, leading to lung immune imbalance and inflammatory response, and affecting the progression of lung diseases. At present, the lung microbiota changes and immune imbalance caused by ectopic colonization of oral microorganisms in the lungs is one of the main ways that oral health status affects respiratory diseases. Studies have shown that *P. gingivalis* in the mouth of periodontitis patients or experimental mice can transfer and colonize on the lungs, causing lung inflammation and injury (16–18). Periodontal pathogens such as *P. gingivalis*, *Fusobacterium. nucleatum*, *Prevotella oralis*, and *Aggregatibacter actinomycetemcomitans* have been identified in the lung aspiration of pneumonia patients (2). Bronchial microbial studies of asthma indicated the presence of four specific bacterial groups, two of which, *Fusobacterium* and *Porphyromonas*, are known periodontal pathogenic bacteria (19). Similarly, higher diversity and rates of microbial colonization were found in the lungs of patients with COPD, and microorganisms from the mouth and upper respiratory tract were detected (2, 20). Previous studies have indicated that the oral periodontitis-associated bacteria *P. gingivalis* acted as one of the main causes of the life-threatening infectious disease aspiration pneumonia and that *P. gingivalis* gingipains could lead to the upregulation of inflammatory factors, including TNF, IL-6, IL 17, and C-reactive protein (21). In short, it indicated that there was a microbial link between periodontitis and COPD state.

Dozens of studies have confirmed that there is, indeed, a positive correlation between periodontitis and COPD (22, 23). People with severe periodontitis have a much higher relative risk of COPD compared with periodontal healthy counterparts (24). The frequency of lung function attenuation and exacerbation in COPD patients with periodontitis is more serious than in COPD patients with good periodontal health (25). Through basic periodontal treatment and oral health maintenance, the frequency of lung function attenuation and exacerbation in COPD patients can be well controlled (26, 27). However, the specific mechanism by which periodontitis affects COPD progression still requires further exploration.

γδ T cells are T cells that perform innate immune functions, which could secret IL 17 and initiate inflammatory responses (28). Studies have shown that oral microorganisms can promote the proliferation of *in situ* γδ T cells in the lungs and secretion of IL 17 to mediate lung inflammation and disease status (29, 30). However, the effect of γδ T cells on COPD is controversial and needs further study. Under the stimulation of IL 4/IL 10/IL 13, macrophages could become M2-polarized, after which cytokines such as MMP9 and MMP12 are produced by M2-polarized macrophages to promote the process of parenchymal injury (31, 32). The occurrence of COPD is related to the enhancement of immune response mediated by macrophage polarization (33, 34). The correlation between M2 polarization and COPD is controversial, and the mechanism of M2 polarization in periodontitis-promoted COPD remains unclear and needs further study. Through chemokines, cytokines, and cell surface receptors, γδ T cells and macrophages may undergo some inter-regulation (35–37). IL 17 and IFN γ produced by γδ T cells can play an inflammatory role; meanwhile, IL 17 can also promote the M2 polarization of lung macrophages (38, 39).

Based on the above background, we hypothesized that in the periodontitis state, the major periodontal pathogenic bacteria, as represented by *P. gingivalis*, may transfer and colonize on the lung tissue, causing microbiota changes in the lung, further leading to pulmonary immune imbalance and promoting the accumulation and proliferation of γδ T cells in the lung. On the one hand, γδ T cells produce IL 17 and IFN γ to directly promote the COPD process; on the other hand, the production of IL 17 by γδ T cells further promotes the M2 polarization of alveolar macrophages and contributes to COPD progression. Therefore, this study intends to determine whether periodontitis promotes COPD progression by regulating γδ T cells and M2 macrophages through a series of experiments, including *in vivo* animal models and *ex vivo* bacterial cell co-culture, and clinical sample detection, in order to provide new directions for clinical COPD patient management and disease control from the perspective of oral health.

## RESULTS

### COPD and periodontitis could mutually promote disease progression

**Fig 1:**
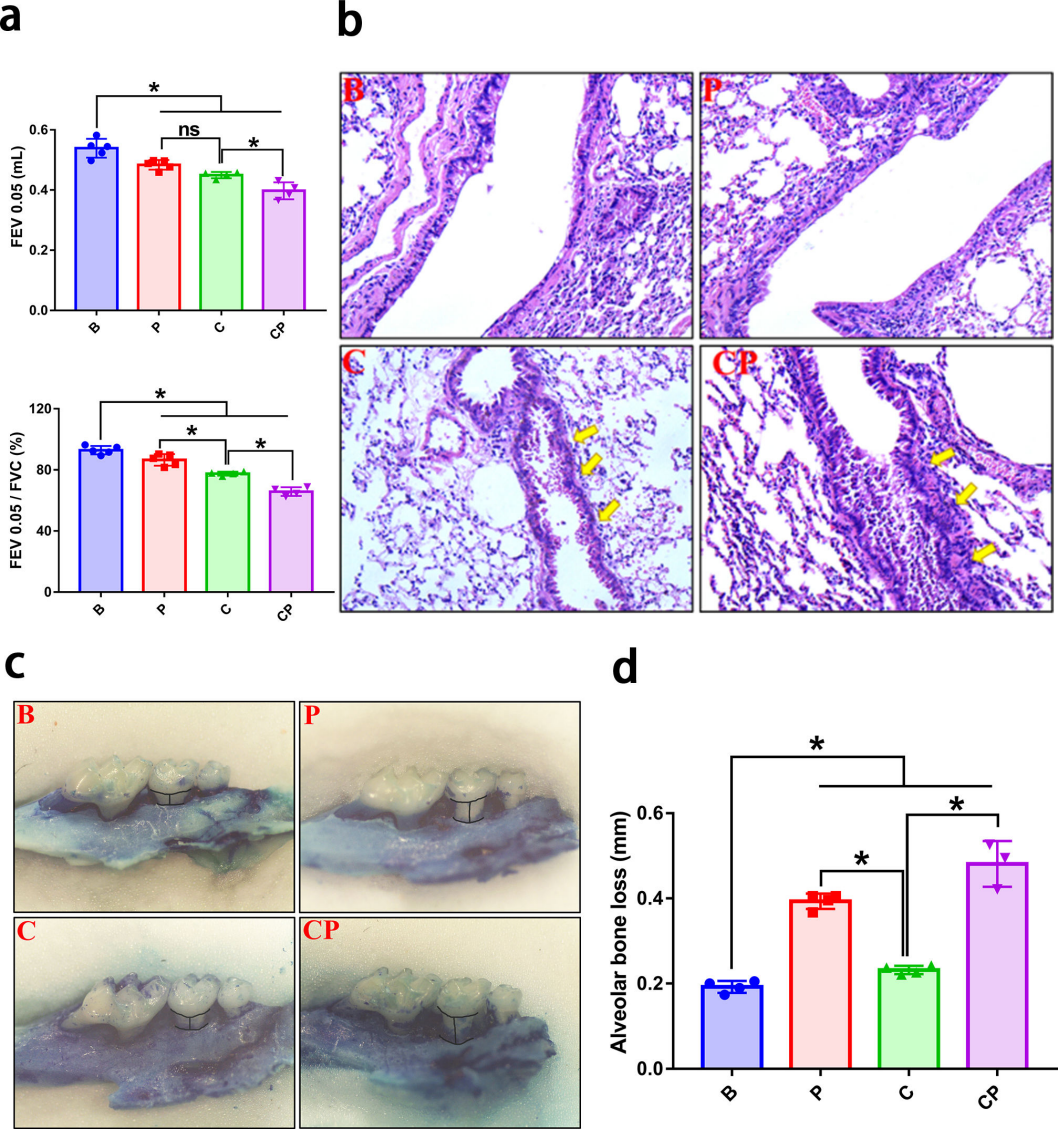
Periodontitis and COPD mutually promoted disease progression. (a) The lung function test was carried out to assess the severity of COPD, and the results were presented with FEV0.05 value and FEV0.05/FVC value for each group. FEV0.05: forced expiratory volume in 0.05 s. FEV0.05/FVC: ratio of forced expiratory volume in 0.05 s to forced vital capacity. (b) H&E stain was performed to observe the lung morphology change and a representative H&E image of lung tissue was shown for each group. (c) The methylene blue stain was carried out to analyze the periodontitis alveolar bone loss and a representative image of the jaw was shown for each group. (d) Quantitative analysis of alveolar bone loss in each group. *P < 0.05. B, blank control; P, periodontitis; C, COPD; CP, COPD with periodontitis.

To observe the effect of periodontitis on COPD progression, we constructed four groups of animal models: B group, blank control group (healthy mice); P group: periodontitis group (mice with periodontitis only); C group, COPD group (mice with COPD only); CP group, COPD with periodontitis group (mice with both COPD and periodontitis). As shown in Fig. 1, periodontitis and COPD could mutually promote disease progression. Periodontitis aggravates the lung function decrease of COPD, with declining forced expiratory volume in 0.05 second (FEV0.05) and ratio of forced expiratory volume in 0.05 second to forced vital capacity (FEV0.05/FVC) (Fig. 1A). H&E observation demonstrated the most seriously dilated and fractured alveolar wall, narrowest airway and most neutrophils infiltration in COPD with periodontitis group (CP group) (Fig. 1B). Similarly, the severity of periodontitis was also most pronounced in the CP group, with the highest amount of alveolar bone resorption (Fig. 1C and D).

### Microbiota homeostasis changed under the COPD and *P. gingivalis*-periodontitis state

**Fig 2:**
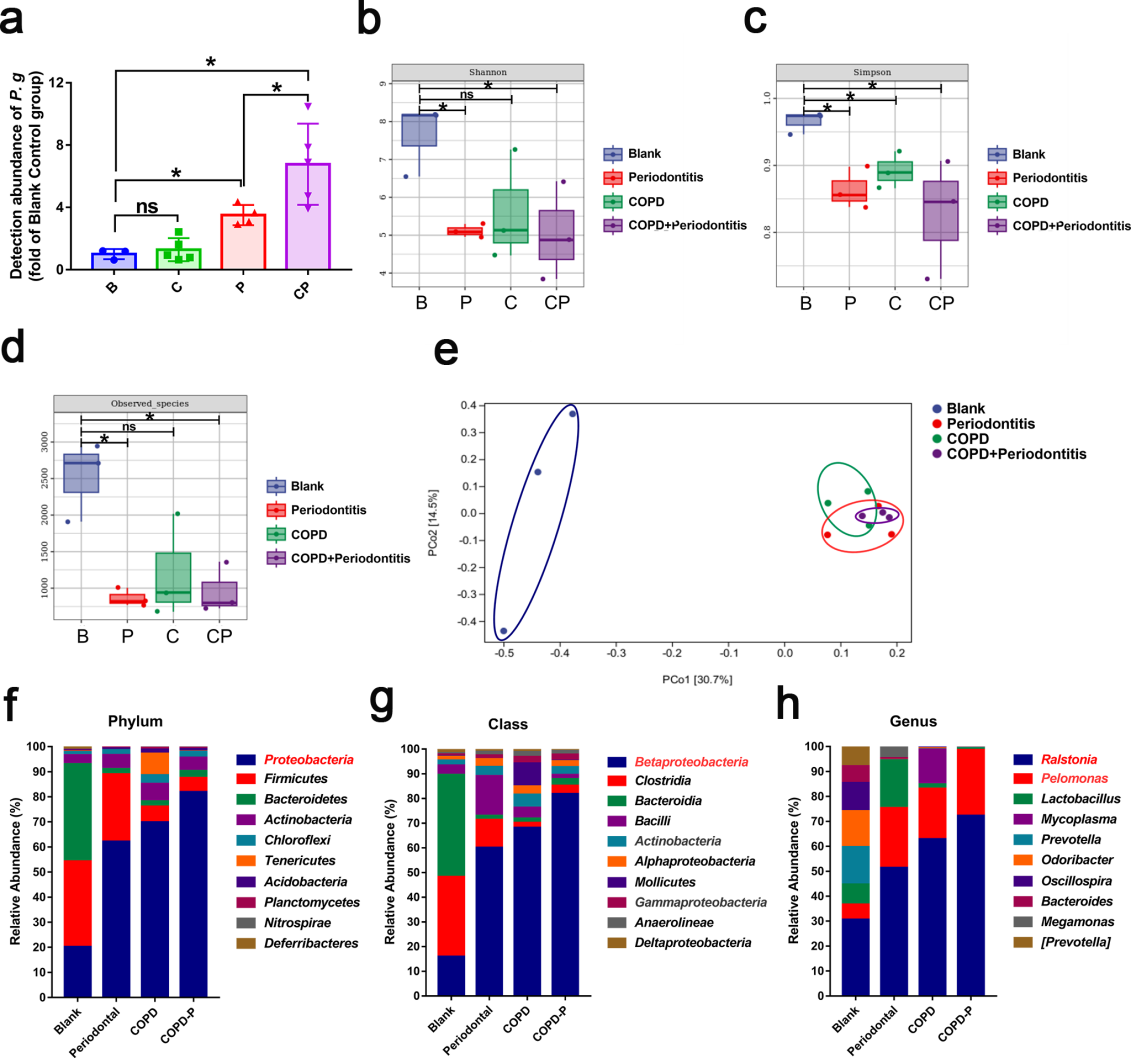
*P. gingivalis*-periodontitis affected homeostasis of the lung microbiota. (a) The relative abundance of *P. gingivalis* in lung tissues was determined by RT-qPCR. (b–d) α-diversity of the lung tissue was analyzed for each group and presented with Shannon, Simpson, and observed species indices. (e) β diversity of the lung tissue in each group was presented with PCOA index. (f–h) Lung microbial community composition was analyzed. The percentages of the major phyla, classes, and genera according to 16S rRNA sequencing were shown. *P < 0.05, ns, not significant; B, blank control; P, periodontitis; C, COPD; CP, COPD with periodontitis.

During the periodontitis model construction procedure, mice were orally infected with *P. gingivalis* (1 × 10^9^ CFU/mL, 0.2 mL/mice) every other day. To analyze whether *P. gingivalis* could migrate to and colonize on the lungs, we assessed the abundance of *P. gingivalis* in the lung tissue and found that periodontitis promoted the increase of *P. gingivalis* abundance in COPD lung tissue (Fig. 2A). To further explore whether *P. gingivalis*-periodontitis caused changes in the microbiota of lung tissue, we carried out 16S rRNA-sequencing analysis. The α diversity indices, β diversity index and differences in the relative abundances of bacterial taxa were compared among the groups to evaluate the microbiota change of lung tissue under different disease states. Through 16S rRNA-sequencing analysis, we observed that the microbiota homeostasis of lung tissue underwent an observable change. Compared to the blank control group (B group), the α diversities, including Shannon, Simpson, and observed species diversity, of the periodontitis (P), COPD (C), and COPD with periodontitis (CP) groups were decreased (Fig. 2B through D); meanwhile, β diversity analysis showed that the P, C, and CP groups showed a different distribution in comparison with the B group (Fig. 2E). Moreover, composition analysis also demonstrated some differences (Fig. 2F through H). Compared to B group (20.25% ± 2.632%), *Proteobacteria* was the most abundant phylum in the P (63.03% ± 18.57%), C (70.47% ± 7.595%), and CP (81.47% ± 5.152%) groups, and in the CP group, the periodontitis condition could further increase the abundance of *Proteobacteria* compared to C group (Fig. 2F). Similarly, at the class level, *Betaproteobacteria* was the most abundant taxon in the P (60.95% ± 17.2%), C (68.71% ± 7.085%), and CP (81.23% ± 5.96%) groups compared to the B group (16.08% ± 2.326%), and periodontitis could further increase the abundance of *Betaproteobacteria* in the COPD lung (Fig. 2G). At the genus level, compared to B group (34.53% ± 15.36%, 7.157% ± 4.387%, respectively), *Ralstonia* and *Pelomonas* were the two most abundant taxa in the P (52.42% ± 15.42%, 25% ± 11.63%, respectively), C (64.51% ± 12.46%, 20.92% ± 5.721%, respectively), and CP (72.15% ± 3.359%, 26.28% ± 4.887%, respectively) groups, and both of these taxa were increased in the CP group compared to C group (Fig. 2H). Interestingly, it was observed that in the CP group, *Proteobacteria* (40) and *Ralstonia* (41), which are associated with lung inflammation, were increased in comparison to the C group. Some oral-associated pathogenic bacteria, including *Lactobacillus*, *Prevotella,* and *Bactriodes,* were also detected in the lung tissues. Compared to C group, some periodontal inflammatory-associated bacteria, including *Bacteroidetes*, *Bacteroides,* and *Prevotella,* showed increased relative abundance in CP group (Table S1). In short, *P. gingivalis*-periodontitis affected the microbiota homeostasis of lung tissue.

### In the development of periodontitis promoting COPD, γδ T cells and polarization of macrophages were activated

**Fig 3:**
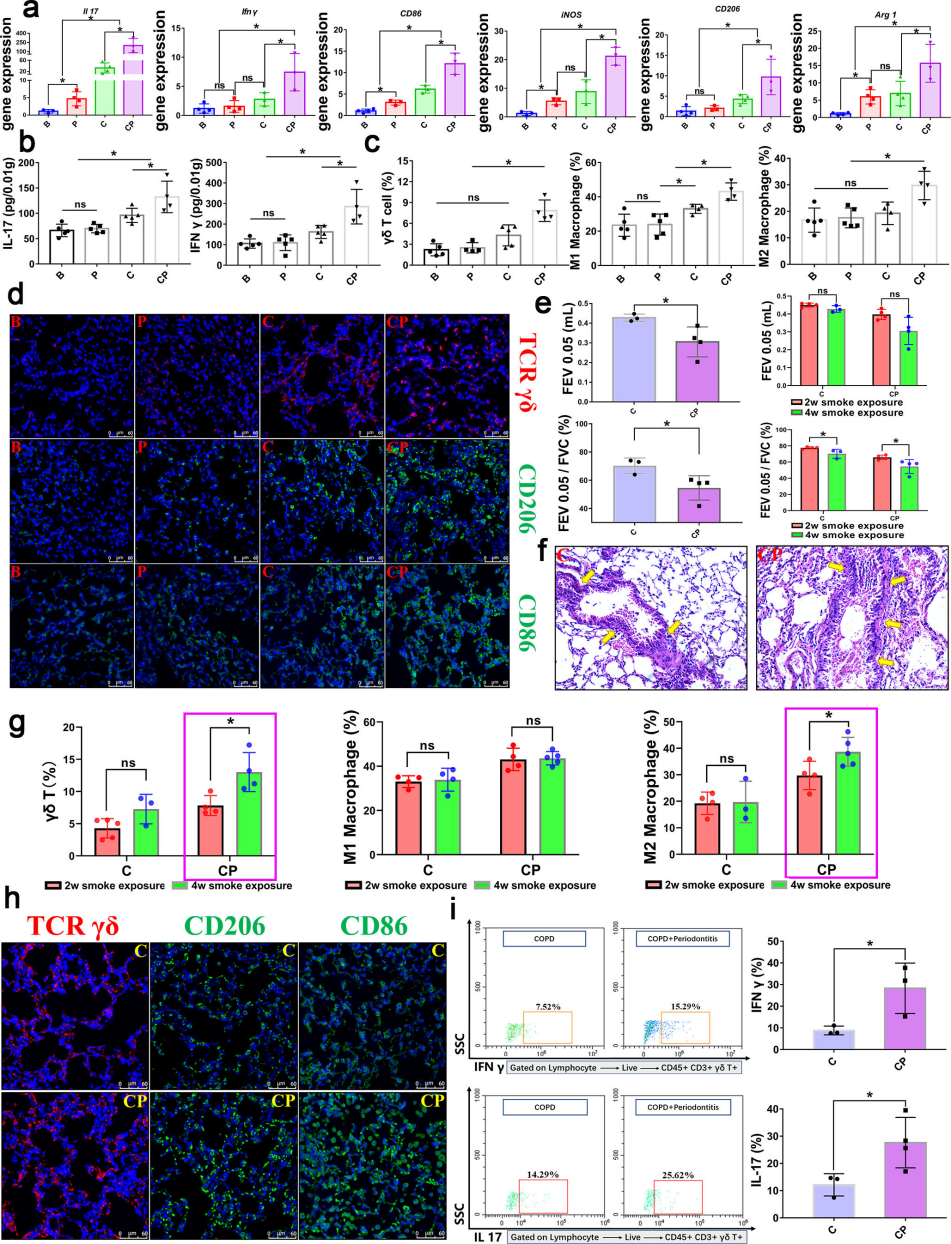
Periodontitis exacerbated COPD through promoting γδ T cells aggregation and macrophages polarization in the lung tissue, and the promotive effect was prolonged over time. (a–d) Results of the early stage of COPD (short smoke exposure time, 2 weeks of daily exposure) model; (e–i) Results of the late stage of COPD (extended smoke exposure time, 4 weeks of daily exposure) model. (a) In the early stage of COPD, the relative mRNA expression of γδ T-related genes, *Il 17* and *Ifn γ*, M1-related genes, *CD86* and *iNOS*, M2-related genes, *CD206* and *Arg 1* in the lung tissue of each group were determined by RT-qPCR and displayed with the fold change to B group. (b) The levels of IL 17 and IFN γ in lung tissue of each group were calculated by ELISA for early stage of COPD. (c, d) In the early stage of COPD, the percentages of γδ T positive cells, M1 polarized macrophages, and M2 polarized macrophages in the lung tissue of each group were quantified by flow cytometry analysis (γδ T cells were gated on the lymphocytes, live cells, and CD45 positive and CD3 positive cells. M1 and M2 cells were gated on all cells, live cell, and CD45 positive and F4/80 positive cells.) and observed by confocal immunofluorescence analysis. Blue color, DAPI; red color, TCR γδ; green color, CD206 or CD86. Following, we further extended the smoke exposure time to 4 weeks and analyzed the effect of periodontitis on COPD in the time-extended (late-stage) model. (e) In the late stage of COPD, the lung function test results of each group and comparison with the corresponding early stage of COPD (2 weeks smoke exposure) were analyzed. FEV0.05, forced expiratory volume in 0.05 s. FEV0.05/FVC: ratio of forced expiratory volume in 0.05 s to forced vital capacity. (f) Representative H&E images of lung tissues in the late stage of COPD (4 weeks smoke exposure) were shown. (g) The quantitative cell numbers calculated by flow cytometry between early stage and late stage of COPD were compared, among which γδ T cells and M2 macrophages showed a significant increase, so, in the following research, we focused on these two types of immune cells. (h) Representative confocal immunofluorescence images of lung tissue in the late stage of COPD. Blue color, DAPI; red color, TCR γδ; green color, CD206 or CD86. (i) In the late stage of COPD, we further observed periodontitis promoted the aggregation of IL 17^+^ γδ T cells and IFN γ^+^ γδ T cells. Representative flow cytometry plots of IL 17^+^ γδ T cells and IFN γ^+^ γδ T cells and corresponding quantitative analysis of lung tissue in late stage of COPD were shown. *P < 0.05, ns, not significant. B, blank control; P, periodontitis; C, COPD; CP, COPD with periodontitis.

To further explore the potential immune mechanism by which periodontitis affected COPD, we analyzed the gene expressions of immune cytokines. In the early stage of COPD (2 weeks of smoke exposure), RT-qPCR results showed that the expression levels of *Il 17* and *Ifn γ*, M1 polarization, and M2 polarization-related markers and inflammatory cytokines (*CD86, iNOS, Il 1β, Il 23, Mmp 9, Mmp 12, Il 4*) in COPD lung tissues were significantly up-regulated under the condition of periodontitis (Fig. 3A; Fig. S1a). ELISA results showed that periodontitis significantly up-regulated the levels of IL 17 and IFN γ in COPD lung tissues (Fig. 3B) and serums (Fig. S1b). These data preliminarily indicated that IL 17 and IFN γ production and macrophage polarization might play important roles in mediating periodontitis-promoting COPD. Considering that γδ T cells possess the ability to produce both IL 17 and IFN γ, we hypothesized that periodontitis might affect COPD progression by promoting the activation of γδ T cells and macrophage polarization. Interestingly, both flow cytometry analysis and immunofluorescence (IF) observation suggested that periodontitis promoted the aggregation and expansion of γδ T cells in COPD lung tissue and, simultaneously, macrophage polarization in CP group lung tissues was significantly increased, including M1 and M2 polarization (Fig. 3C and D; Fig. S1c). These data preliminarily suggested that periodontitis could promote COPD progression through the activation of γδ T cells and polarization of macrophages.

### The promotive effect of periodontitis on COPD progression through γδ T cells and M2 macrophages was prolonged over time

Further extending smoke exposure time by another 2 weeks (4 weeks of smoke exposure, totally), in the later stages of COPD, we found that the mutually reinforcing interaction between periodontitis and COPD was more pronounced in the mouse models with the extended smoke exposure time (Fig. 3E and F). In CP group, lung function was further decreased, with further decreases in FEV0.05 value and FEV0.05/FVC (Fig. 3E). Lung tissue lesions were more obvious; airway inflammatory infiltration was aggravated (Fig. 3F). Similarly, alveolar bone resorption was also further aggravated (Fig. S3a).

Gene expression analysis showed that periodontitis still up-regulated the gene expression of *Il 17*, *IFN γ*, and macrophage polarization-related markers and inflammatory cytokines in COPD lung tissue in the later stage (Fig. S2a). ELISA analysis also confirmed that periodontitis up-regulated the levels of IL 17 and IFN γ in COPD lung tissue and serum and that the up-regulation effects were significantly stronger than those of the early stage (Fig. S2b and c).

Flow cytometry analysis and IF observation also showed that periodontitis further promoted the aggregation and proliferation of γδ T cells and the polarization of macrophages in lung tissue in the CP group (Fig. 3G and H; Fig. S3b). Among them, periodontitis significantly increased the γδ T cells and M2 macrophage polarization in COPD lung tissue compared to the earlier stage model, while the M1 polarization did not change significantly (Fig. 3G). Therefore, we speculated that periodontitis played an important role in promoting the progress of COPD mainly through the up-regulated γδ T activation and M2 polarization, and in the following experiments, we focused on the role of γδ T activation and M2 polarization. Further analysis showed that periodontitis could further up-regulate the ability of γδ T cells to express IL 17 and IFN γ in lung tissue during the later stage of COPD (Fig. 3I). These results indicated that the promotive effect of periodontitis on COPD progression was prolonged over time and activation of IL 17^+^ γδ T cells, IFN γ^+^ γδ T cells, and M2 macrophages played important roles in how periodontitis affected COPD progression.

### The periodontitis-associated bacteria *P. gingivalis* could activate γδ T cells and M2 macrophages

**Fig 4:**
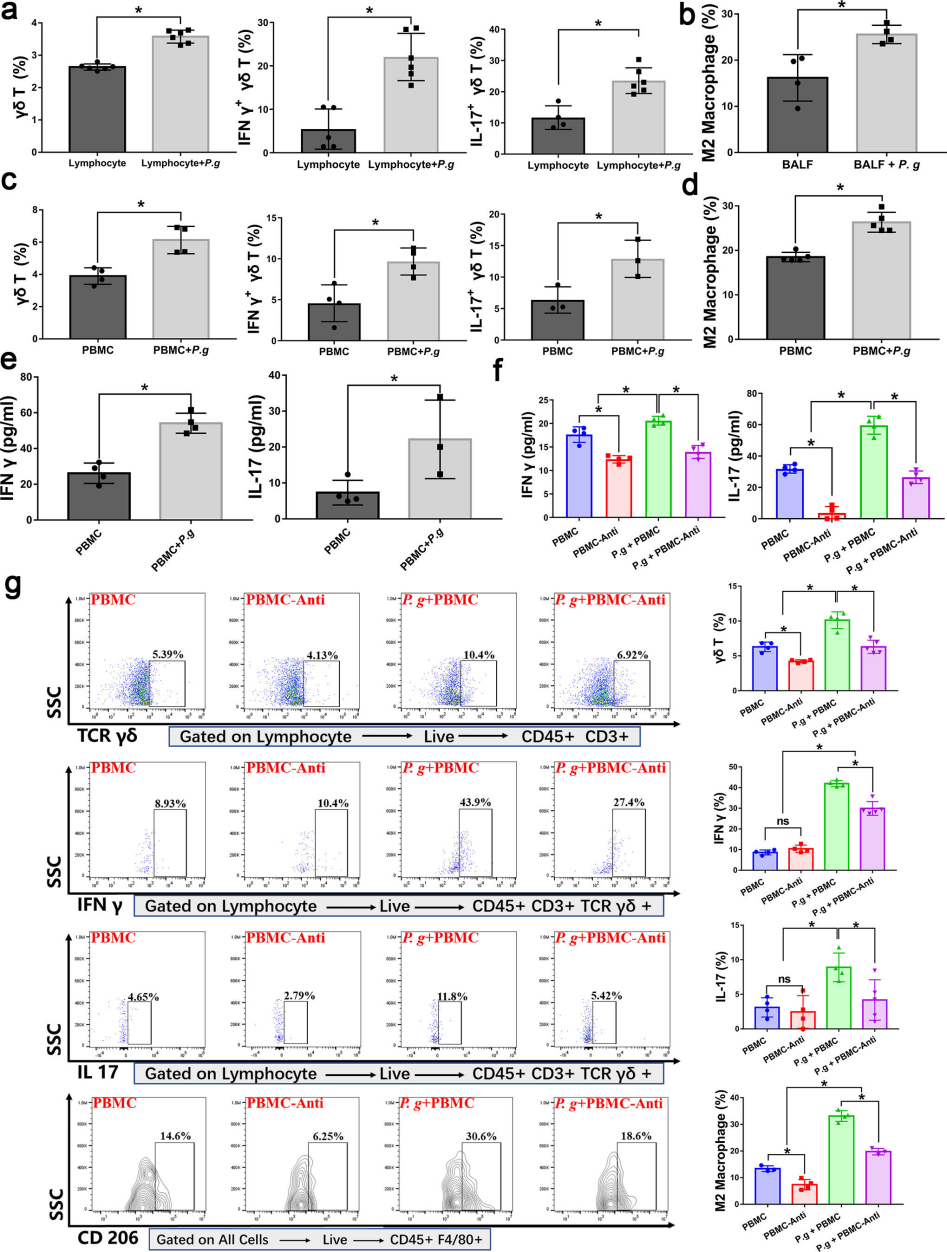
Periodontitis-associated bacteria *P. gingivalis* promoted γδ T cells activation and M2 polarization in the lung tissue and PBMC. The promotion effects were affected by γδ-TCR monoclonal antibody treatment. These *ex vivo* experiments were conducted using mice materials, including mice lung tissue lymphocytes, BALF and PBMC. (a, b) *P. gingivalis* promoted γδ T cells activation in lung tissue lymphocytes, and M2 macrophages in the BALF, the percentages of γδ T cells, IFN γ^+^ γδ T cells, IL 17^+^ γδ T cells (a) and M2 macrophages (b) were calculated. (c, d) *P. gingivalis* promoted γδ T cells activation and M2 polarization in the PBMC, the percentages of γδ T cells, IFN γ^+^ γδ T cells, IL 17^+^ γδ T cells (c) and M2 macrophages (d) were calculated. (e, f) *P. gingivalis* promoted the levels of IL 17 and IFN γ in the PBMC supernatants (e), and the promotion effect was impacted by γδ-TCR monoclonal antibody treatment (f). (g) With or without γδ-TCR monoclonal antibody treatment, *P. gingivalis* were cocultured with PBMC. γδ T cells activation and M2 polarization were analyzed by flow cytometry and representative flow cytometry plots were shown, and the corresponding quantitative analysis of γδ T cells, IL 17^+^ γδ T cells, IFN γ^+^ γδ T cells, and M2 cells was presented as mean ± STD. *P < 0.05, ns, not significant. Lung-lym, the lymphocytes of lung tissue; Lung-lym +*P. g,* the lymphocytes of lung tissue cocultured with *P. gingivalis;* BALF, bronchoalveolar lavage fluid; BALF +*P. g,* bronchoalveolar lavage fluid cocultured with *P. gingivalis;* PBMC, peripheral blood mononuclear cells; PBMC +*P. g*, peripheral blood mononuclear cells cocultured with *P. gingivalis;* PBMC-Anti, PBMC group with γδ-TCR monoclonal antibody treatment; *P. g* + PBMC-Anti, *P. g* + PBMC group with γδ-TCR monoclonal antibody treatment.

To evaluate whether the periodontitis-associated bacteria *P. gingivalis* had the potential to activate γδ T cells and M2 macrophages, we carried out a series of *ex vivo* experiments. These *ex vivo* experiments were conducted using mice materials, including mice lung tissue lymphocytes, BALF, and peripheral blood mononuclear cells (PBMC). As shown in Fig. 4; Fig. S4, P. *gingivalis* could stimulate the γδ T cells activation in lung lymphocytes and promote their ability to produce IL 17 and IFN γ (Fig. 4A; Fig. S4a). In addition, *P. gingivalis* could also stimulate the M1 and M2 polarization of macrophages in lung BALF (Fig. 4B; Fig. S4a through S4c). Furthermore, in the PBMC environment, co-coculture with *P. gingivalis* elevated γδ T cells, IL 17^+^ γδ T cells, and IFN γ^+^ γδ T cells (Fig. 4C; Fig. S4b), M1 polarized macrophages and M2 polarized macrophages (Fig. 4D; Fig. S4b through S4d), as well as the levels of IL 17 and IFN γ levels in the supernatants (Fig. 4E). To further confirm the important role of γδ T cells, we carried out γδ T cells inhibition treatment. Under the inhibitor-treated PBMC environment (the γδ-TCR monoclonal antibodies were first intraperitoneally injected *in vivo*, and 24 h later, the peripheral blood of mice was collected to obtain the inhibitor-treated PBMC), we observed that the levels of IL 17 and IFN γ in the supernatants were significantly decreased (Fig. 4F);also, the activation potentials of *P. gingivalis* on γδ T cells and M2 macrophage polarization were significantly decreased (Fig. 4G). While, under γδ-TCR monoclonal antibody treatment, the M1 polarization of macrophages was not significantly affected, regardless of the presence or absence of *P. gingivalis* stimulation (Fig. S5). These results indicated that *P. gingivalis* could activate γδ T cells activation and M2 macrophage polarization and that the M2 macrophage polarization was affected by the γδ T cells.

Therefore, combined with the above experimental results, we hypothesized that periodontitis could aggravate COPD through the γδ T-M2 immune mechanism. On the one hand, periodontitis promoted γδ T cells aggregation and proliferation and the production of IL 17 and IFN γ in lung tissue, which could aggravate COPD progression directly; on the other hand, the activated γδ T cells mediated M2 polarization of macrophages (IL 17^+^ γδ T cells in the lung tissue possessed the potential to induce M2 polarization of macrophages), M2 macrophages could further aggravate COPD progression.

### Inhibition of γδ T cells affected the promotion of COPD by periodontitis

**Fig 5:**
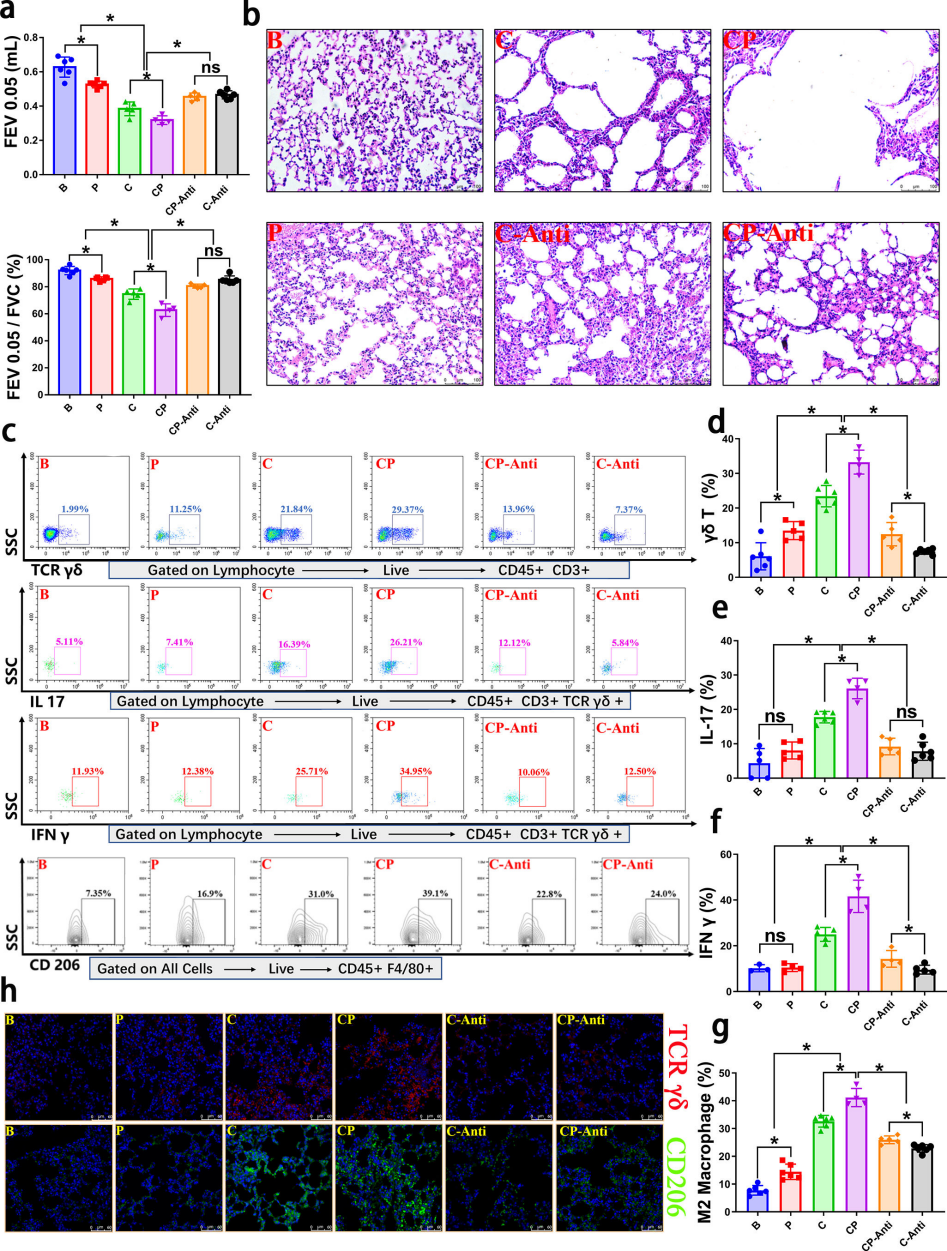
Inhibition of γδ T cells affected periodontitis promoting COPD *in vivo*. (a) With or without the γδ-TCR monoclonal antibody treatment, the lung function test was performed in each group, and the results were shown as FEV0.05 value and FEV0.05/FVC value. FEV0.05, forced expiratory volume in 0.05 s. FEV0.05/FVC, ratio of forced expiratory volume in 0.05 s to forced vital capacity. (b) A representative H&E image of lung tissue in each group was shown. Periodontitis aggravated COPD, while γδ-TCR monoclonal antibody treatment partly reduced COPD severity. (c–g): Representative flow cytometry plots and corresponding quantitative analysis of lung tissue for each group were shown. (h) With or without γδ-TCR monoclonal antibody treatment, representative confocal immunofluorescence images of γδ T cells and M2 macrophages of lung tissue for each group were shown. Blue color, DAPI; red color, TCR γδ; green color, CD206. *P < 0.05, ns, not significant. B, blank control; P, periodontitis; C, COPD; CP, COPD with periodontitis; C-Anti, the COPD group with γδ-TCR monoclonal antibody treatment; CP-Anti, the CP group with γδ-TCR monoclonal antibody treatment.

To further confirm the essential effect of γδ T activation in periodontitis’ influence on COPD progression and M2 polarization *in vivo*, we constructed mouse models with γδ-TCR monoclonal antibody inhibition treatment. As shown in Fig. 5, in the anti-γδ T groups (C-Anti group and CP-Anti group), lung functions and lung lesions were significantly improved compared to those of the C and CP groups, which phenotypically confirmed the important role of γδ T cells in how periodontitis affects COPD (Fig. 5A and B). However, alveolar bone resorption was not significantly affected by the γδ-TCR monoclonal antibody inhibition treatment (Fig. S6).

Flow cytometry and immunofluorescence observation verified the significant reduction of γδ T cells expansion in the lung tissues in the anti-γδ T groups (C-Anti group and CP-Anti group) compared to the groups without γδ-TCR monoclonal antibody treatment (C group and CP group, respectively); meanwhile, compared to C group and CP group, COPD severity in C-Anti group and CP-Anti group was significantly improved, respectively. These results indicated that γδ-TCR monoclonal antibody treatment was associated with milder COPD symptoms. Interestingly, inhibition of the γδ T cells also resulted in a decrease of M2 macrophages in the lung tissue (Fig. 5C, G and H). Consistent with the *ex vivo* results, under γδ-TCR monoclonal antibody treatment, the M1 polarization of macrophages in the lungs was not significantly affected, regardless of the presence or absence of periodontitis (Fig. S7). These results again demonstrated that the activation of γδ T cells in lung tissue partly induced the M2 polarization of macrophages and promoted disease progression in the periodontitis-promoted COPD model (Fig. 5C through G). Similarly, the ELISA and RT-qPCR results indicated that γδ-TCR monoclonal antibody treatment decreased the levels of IL 17 and IFN γ in lung tissue (Fig. S8a) and serum (Fig. S8b), as well as gene expression levels in lung tissue (Fig. S8c and d).

Briefly, these data suggested that activation of γδ T cells played a crucial role in both M2 macrophage polarization and COPD progression. M2 macrophages acted as another immune-based cause of COPD progression and were affected by γδ T cells in this study. Therefore, it could be concluded that in the current periodontitis-promoted COPD model, activation of γδ T cells affected M2 macrophage polarization and periodontitis exacerbated COPD through the promotion of the γδ T-M2 immune mechanism.

### Enhanced IFN γ and IL 17 levels and M2 polarized gene expressions in clinical COPD with periodontal pathogenic bacteria BALF samples

**Fig 6:**
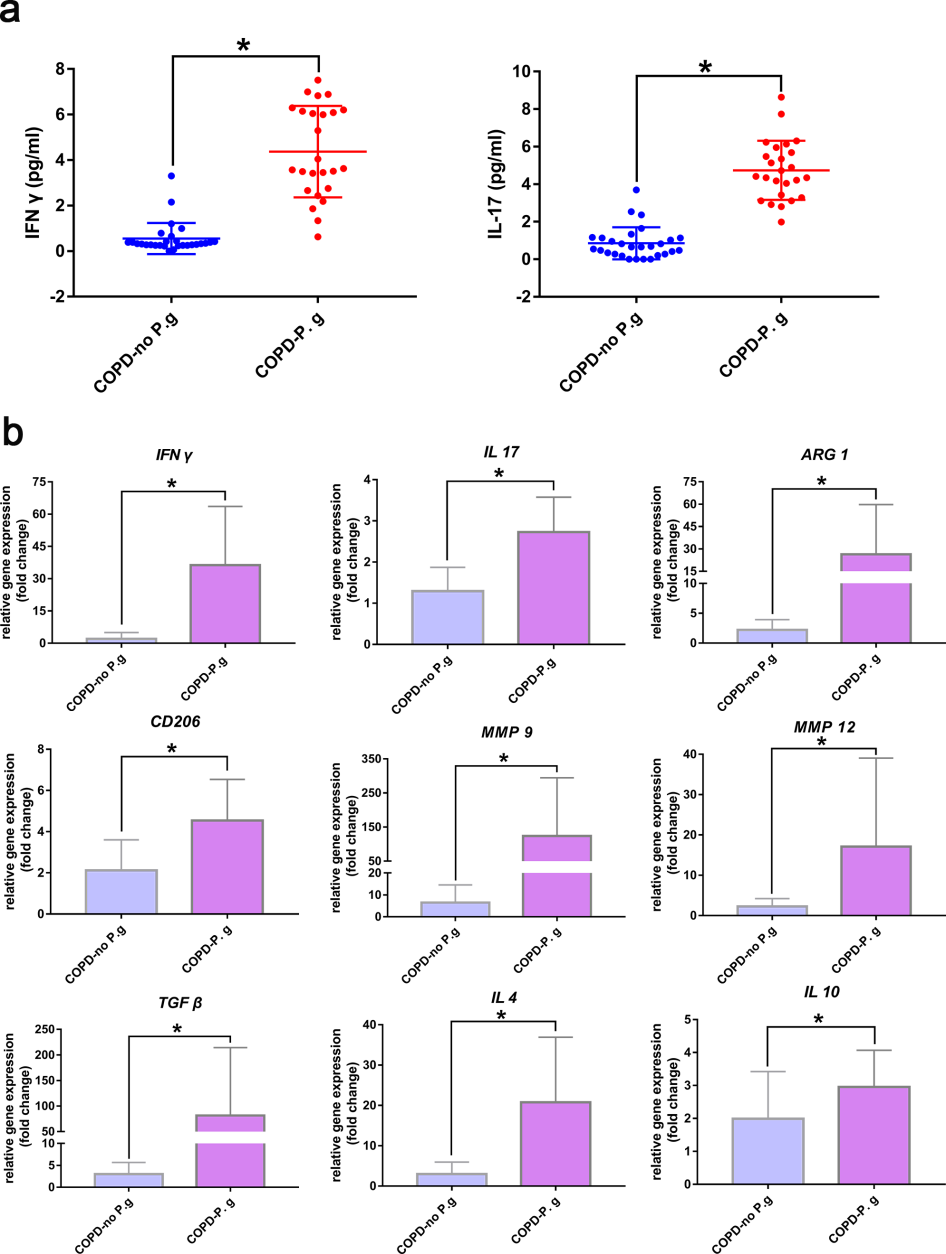
Enhanced IFN γ and IL 17 levels and expression of M2-polarized genes in clinical COPD with periodontal pathogen BALF samples. (a) IFN γ and IL 17 levels in clinical COPD BALF samples were measured by ELISA. (b) Relative gene expression levels in clinical COPD BALF samples were quantified by RT-qPCR. *P < 0.05, ns, not significant. COPD-no *P. g*, BALF of COPD patients with no *P. gingivalis* detected; COPD-*P. g*, BALF of COPD patients with *P. gingivalis* detected.

The data presented above demonstrated that periodontitis aggravated COPD progression through activation of γδ T cells and M2 macrophages. The expression of IL 17 and IFN γ by activated γδ T cells directly promoted COPD, and, simultaneously, the expressed IL 17 induced M2 polarization of macrophages, which also promoted COPD. To further confirm the important roles of IFN γ and IL 17 levels and M2-polarized gene expressions in clinical COPD with periodontal pathogenic bacteria BALF samples, 53 clinical BALF samples were collected. After *P. gingivalis* detection analysis, 25 samples that were positive for the presence of *P. gingivalis* were assigned to the COPD-*P. g* group, and the other 28 samples that lacked *P. gingivalis* were assigned to the control group (COPD-no P.g). The characteristics of clinical samples in the two groups were shown in Table S2. The systemic conditions of the patients in the two groups were consistent, and there were no significant differences in age, gender, lifestyle habits such as smoking, systemic disease conditions such as hypertension, diabetes, etc. We further reviewed the COPD disease status of the sampled patients, and the results showed that among the 25 subjects with *P. gingivalis* detection, 20 samples were diagnosed as being in the acute exacerbation stage, while among the 28 samples in the control group, 19 were in the acute exacerbation stage of COPD. That was to say, the incidence of acute exacerbation of COPD in the experimental group (80.00%) was higher than that in the control group (67.86%), which could reflect to a certain extent that the existence of periodontal pathogenic bacteria was related to the exacerbation of COPD. ELISA measurements suggested that higher levels of IL 17 and IFN γ in the BALF supernatants of COPD with periodontal pathogenic bacteria (*P. gingivalis*) (Fig. 6A). Gene expression analysis also indicated that the expression levels of IL 17/IFN γ and M2 related genes *ARG1*, *CD206*, *MMP9*, *MMP12*, *TGF β*, *IL 4,* and *IL10* were higher in COPD BALF samples with the presence of *P. gingivalis* (Fig. 6B). Our results demonstrated that in the clinical environment, IFN γ and IL 17 levels and M2 polarized genes might be important factors in COPD with periodontitis.

## DISCUSSION

Periodontitis is a chronic inflammatory disease of the periodontal tissue caused by plaque biofilm, which acts as a potential risk factor for a variety of systemic diseases, including cardiovascular disease, diabetes, hypertension, various cancers, COPD, etc. (5, 42, 43). As is commonly known, globally, chronic respiratory diseases act as the leading cause of death and morbidity, among which COPD causes the most deaths, and is considered the third leading cause of death worldwide (44). COPD is a devastating lung disease characterized by an incomplete and reversible airflow restriction that is usually progressive and associated with the lungs' abnormal inflammatory response to harmful particles or gases (45). Many previously published studies reported the positive association between periodontitis and COPD (22, 23), but the potential immune mechanisms were unclear.

In the current study, we found that periodontitis could aggravate COPD progression (Fig. 1), which was consistent with previous studies (1, 46). Further microbe analysis indicated that microbiota composition changed under disease state, and that periodontitis caused an increase of lung inflammation associated bacteria, such as *Proteobacteria* and *Ralstonia*. To some degree, this phenomenon indicated that periodontitis could promote lung inflammation development. Like previous studies (2, 19, 20), we also observed the microorganism communication between lung tissue and the oral cavity. Many oral bacteria were detected in the lung tissues, including *Lactobacillus, Prevotella*, *Bactriodes,* and *P. gingivalis*. Periodontitis also caused the increase of two main bacterial taxa, *Ralstonia* and *Pelomonas*, in the COPD lungs. Moreover, some oral periodontal-associated pathogenic bacteria, including *Prevotella*, *Bactriodes,* and *P. gingivalis* were also detected with increased relative abundance in the COPD with periodontitis lung tissue compared to those in the COPD group. Therefore, it could be concluded that periodontitis affected the microbiota homeostasis of the lung.

γδ T cells perform innate immune functions and act as an important subset of T cells, which play an important role in inflammatory immunity via the secretion of cytokines, including IFN γ and IL 17 (28). IL 17 is a pivotal cytokine that regulates lung immunity and inflammation and has shown the capacity to mediate COPD progression (47, 48). IFN γ is another crucial cytokine that is associated with COPD. Numerous studies demonstrated that the IFN γ levels as well as IFN γ-producing cells were increased in COPD patients and experimental mouse models, while effective COPD treatment resulted in decreased IFN γ levels (49–51). Moreover, a recent study indicated that Th17/Th1 cells capable of secreting both IL 17 and IFN γ were more pathogenic in COPD (52).

In lung tissue, γδ T cells constitute a major tissue-resident T cell component and play important roles in maintaining the homeostasis of lung tissue (53, 54). The effects of γδ T cells have two sides in lung tissue. Some studies have indicated that γδ T cells helped to activate host defense against bacterial, viral, and fungal infections and participated in maintaining lung tissue homeostasis (53, 54). Under inflammation state of lung tissue, γδ T cells displayed a tissue reparative effect through the production of IL 17 and were found to be associated with inflammation control (54, 55). Additionally, γδ T cells in lung tissue also showed protective effects towards cystic fibrosis and lung cancers (53, 56, 57). However, on the contrary, numerous other studies have indicated that γδ T cells might act with a crucial pathogenic effect in lung diseases. γδ T cells and their IL 17 expression are required for pulmonary inflammation and injury caused by ozone exposure (30). IL 17A-producing γδ T and Th17 lymphocytes mediated lung inflammation and acute lung injury in experimental silicosis (58). Jin et al. (29) implicated that commensal-bacteria activated γδ T cells in lung tissue and promoted lung adenocarcinoma through IL 17 production. Data in Vigeland’s study elucidate the critical role of IL 17A^+^ γδ T cells in promoting chronic inflammation and fibrosis of the lung tissue (39).

Similarly, there has been some controversy about the role of γδ T cells in COPD progression. Pons et al. (59) reported blunted γδ T-lymphocyte response in the COPD disease state. Similarly, Urboniene et al. (60) investigated γδ T cells in induced sputum (IS) and bronchoalveolar lavage (BAL) and found reduced amount of γδ T cells in IS and BALF from COPD patients compared those from asthmatic or healthy subjects, which indicated that γδ T cells might have a protective effect in preventing COPD. However, inconsistent with this, Majo et al. observed in clinical lung tissue samples that γδ T cells significantly increased in the lungs of smoker and emphysema patients (61). Smoking causes or exacerbates COPD by promoting γδ T proliferation and IL 17 production (48). Serum amyloid A promotes lung neutrophil accumulation and COPD by increasing γδ T cells and the associated expression of IL 17 (62). In this study, we found that γδ T cells played an important role in the cigarette smoke exposure-induced COPD disease model, suggesting that smoking led to the expansion of γδ T cells in lung tissue and contributed to the occurrence of COPD (Fig. 3 and 5), consistent with Majo’s and Bozinovski’s results (48, 61). More importantly, we further found that γδ T cells were important targets for mediating the promotive effect of periodontitis on COPD. In the presence of periodontitis, the severity of COPD disease was much greater, the proportion of γδ T/IL17^+^ γδ T/IFN γ^+^ γδ T cells in COPD lung tissue was significantly up-regulated, and the contents of IL 17 and IFN γ were also significantly increased (Fig. 3; Fig. S1 and S2). However, when γδ T cells were inhibited, the proportion of γδ T/IL 17^+^ γδ T/IFN γ^+^ γδ T cells in lung tissue was significantly reduced, the contents of IL 17 and IFN γ were decreased, the severity of COPD disease was significantly decreased, and the promotive effect of periodontitis on COPD was also significantly affected (Fig. 5; Fig. S8). Therefore, these results suggested that periodontitis contributed to COPD progression by promoting γδ T cells activation in COPD lung tissue.

Macrophages are innate immune cells and play important roles in homeostasis and host defense. The development of COPD is associated with enhanced immune response mediated by macrophage polarization (33, 34, 63, 64). A majority of alveolar macrophages are normally non-polarized in healthy lung tissue, but in lung tissue of COPD, there is a significant increase of macrophage polarization and co-expression of M1 and M2 macrophages (65, 66). Moreover, both M1-polarized and M2-polarized macrophages in the lung tissue increased significantly with the progression of smoke and COPD severity (65). However, the role of distinct macrophage phenotypes (M1 versus M2) in COPD is unclear and which macrophage phenotype (M1 versus M2) is more predominant in COPD progression is still controversial. Through literature review, Lee et al. (64) concluded that M1-associated marker (iNOS) and cytokines (IL-1β, IL-6, IL-8, TNF-α) were increased in COPD patients, indicating the pathogenic effect of M1 polarization in COPD. LncRNA MIR155HG promoted COPD by upregulating M1 polarization and downregulating M2 polarization of lung macrophages (67). Similarly, Sun’s data indicated ergosterol showed therapeutic effect toward COPD by decreasing M1 polarization and increasing M2 polarization (68). While, dozens of studies have pointed out that M2 polarization of macrophages was the essential pathogenesis of COPD. Eapen et al. (69) observed that in COPD patients, luminal macrophages showed a dominant polarization of M2 phenotype and BALF showed increased cytokines of the M2 profile, including CCL22, IL-4, IL-13, and IL-10. Liu et al. (70) emphasized the important role of M2 polarization of macrophage and suggested that through suppressing M2 macrophage polarization, effective components combination (ECC) could improve airway remodeling of COPD. In the current study, we found that periodontitis promoted macrophage polarization in COPD lung tissue, including M1 polarization and M2 polarization, and the effect of periodontitis on M2 polarization in COPD lung tissue was significantly enhanced with the disease duration, which is consistent with the study of Feng et al. (71), while, M1 polarization did not change significantly with the disease duration. Therefore, we assumed that in the later stage of the disease, M2 polarization played an important role in mediating the promotive effect of periodontitis on COPD.

Generally, IL 4, IL10, and IL 13 are considered the more common markers for inducing M2 polarization; however, existing studies have shown that in the lung tissue, γδ T cells could regulate M2 polarization of macrophages by producing IL17 and that M2 polarization could be significantly inhibited if γδ T cells was deficient or IL17 was blocked. In lung tissue, IL 17 expressed by activated γδ T cells could induce macrophage proliferation and M2 polarization, thus further affect disease progression (30, 38, 39). This procedure had been demonstrated in both pulmonary inflammation and lung fibrosis (38, 39) as well as in OSCC with periodontitis models (35). Therefore, does such an immune regulatory mechanism also exist in the COPD disease model and can periodontitis further promote COPD progression by regulating this mechanism? In the current study, we found that M2 polarization was influenced by the activation of γδ T cells in lung tissues in COPD with periodontitis. The increase of γδ T and IL17^+^ γδ T cells could further induce the increase of M2 polarization (Fig. 4 and 5). Similarly, under the condition of γδ T cells inhibition treatment, γδ T and IL17^+^ γδ T cells were significantly reduced, and M2 polarization was also significantly decreased (Fig. 4 and 5). While, under γδ-TCR monoclonal antibody treatment, M1 polarization of macrophages in the lungs was not significantly affected, regardless of the presence or absence of periodontitis (Fig. S7). These results again demonstrated that the activation of γδ T cells in the lung tissue could partly induce the M2 polarization of macrophages and promote disease progression in the periodontitis-promoted COPD model. Importantly, in clinical BALF samples, we obtained similar results. In COPD BALF samples containing the periodontal pathogenic bacteria *P. gingivalis*, the levels of IL 17 and IFN γ and the gene expressions of *IL 17*, *IFN γ,* and M2 polarization associated genes, including *ARG1*, *CD206*, *MMP9*, *MMP12*, *TGF β*, *IL 4,* and *IL10*, were significantly upregulated (Fig. 6), indicating the important roles of IL 17 and IFN γ and M2 macrophages in periodontitis-aggravated COPD in the clinical samples. While, in the disease models of current research, the regulatory mechanism of M1 polarization may have a more complex regulatory network, which needs to be further explored in the future. In the current disease model, periodontitis promoted the activation of γδ T cells in the lung. On the one hand, IFN γ and IL17 produced by activated γδ T cells directly promoted the progression of COPD. On the other hand, IL17 produced by activated γδ T cells further promoted M2 polarization, thereby aggravating the progression of COPD. However, in this disease model, M1 polarization might be regulated by a variety of inflammatory and immune factors, not just IFN γ. And in our future studies, we will further explore the regulatory network of M1 polarization in this disease model. Moreover, a novel concept mentioned that some novel markers (*CD38*, *Gpr18,* and *Fpr2* for M1 polarization, *Egr2* and *c-Myc* for M2 polarization) (72) were recommended to be contained in the macrophage polarization-associated studies, and in the future’s experimental study, we will further contain these markers.

**Fig 7:**
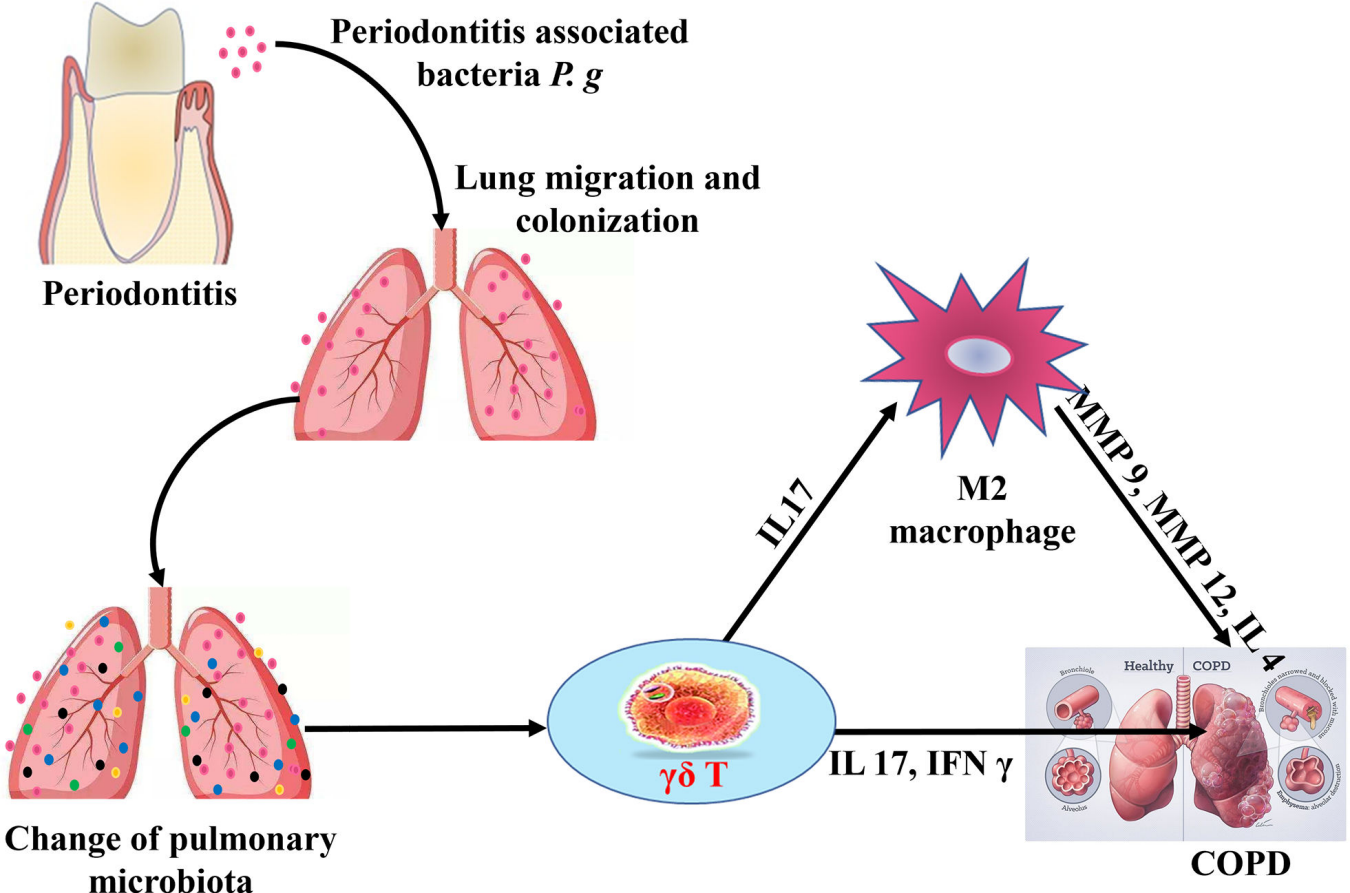
The potential immune mechanism by which periodontitis aggravates COPD progression. Under periodontitis state, periodontitis-associated bacteria, as represented by *P. gingivalis*, migrate to and colonize on lung tissue and affect lung microbiota homeostasis. Following, periodontitis promotes the aggregation and activation of γδ T cells, with IL 17 and IFN γ directly promoting the progression of COPD. Simultaneously, IL 17 further induces M2 polarization of macrophages, thereby contributing to COPD progression.

Through the *in vivo*, *ex vivo,* and clinical samples analysis, we could conclude that in COPD with periodontitis, the microbiota homeostasis of lung tissue changed, and at the same time, an immune imbalance of lung tissue occurred. Periodontitis promoted the activation of γδ T cells, leading to γδ T cells, IL17^+^ γδ T cells, and IFN γ^+^ γδ T cells expansion in the COPD lung tissue. On the one hand, IL 17 and IFN γ directly promoted the progression of COPD, and, simultaneously, IL 17 further induced M2 polarization of macrophages, thereby contributing to COPD progression (Fig. 7).

However, there are some limitations in this research. The first one is the short duration of the later-stage model. In this study, the later-stage model (4-week model) only extended the cigarette smoke exposure time by 2 weeks compared to the earlier-stage model (2-week model), which might be related to the fact that in this research, the decrease of the lung functions and the increase of some immune cells (γδ T cells and M2 macrophages) were modest. If the cigarette smoke exposure could be extended for a longer period of time, these changes in the later-stage model might be more pronounced. In our upcoming research, we will further explore the changes of disease severity and investigate the increases of γδ T cells and M2 macrophages with the animal models of many other longer disease duration-time points. The second limitation is the imbalance between the groups in the proportion of patients in acute exacerbation stage. If the acute exacerbation status of the sampled patients between the two groups was the same, it could be better emphasis the role of periodontal pathogenic bacteria in up-regulating the inflammatory gene expression and inflammatory cytokines content in the lung. And in our forthcoming study, we will further overcome the difficulty of sample collection and obtain the samples without such limitation, to more accurately reflect the difference between the detection of periodontal pathogenic bacteria and the patient’s lung function status.

In summary, we observed that periodontitis could exacerbate COPD progression and identified for the first time that the γδ T-M2 immune mechanism played an important role in mediating periodontitis promoting COPD. The presence of periodontitis intensified the activation of γδ T cells and M2 macrophages in COPD lung tissue, resulting in the aggravation of COPD severity. Therefore, targeting at periodontitis treatment and the γδ T-M2 immune mechanism might be a new practical strategy for the prevention or control of COPD.

## MATERIALS AND METHODS

### Culture of *P. gingivalis*

*P. gingivalis* W83 was grown anaerobically (37°C, 85% N_2_, 10% H_2_, 5% CO_2_). Cells (1 × 10^9^ CFU/mL) were collected for following use. For mouse oral infection, the bacteria were centrifuged and resuspended with 2% carboxymethylcellulose (CMC) solution. For the bacteria-cell co-culture experiments, the bacteria were centrifuged and resuspended in 10% fetal bovine serum (FBS) containing DMEM. The bacteria were then co-cultured with cells at a multiplicity of infection (MOI) of 100 (35, 73).

### Experimental animal model

Mouse models were constructed to study the promotion ability and possible immune mechanism that periodontitis affected COPD. The animal model experiment was approved by the animal research committee of West China School of Stomatology, Sichuan University (WCHSIRB-D-2020-127). Male 7-week-old-specific pathogen free (SPF) C57BL/6J mice purchased from Dashuo Biological Technology (Chengdu, China) were randomly divided into several groups (5 mice/group). For periodontitis construction, the 5-0 silk ligatures were tied around the maxillary second molars of mice. Following, mice were infected with *P. gingivalis* (1 × 10^9^ CFU/mL, 0.2 mL/mice) every other day. Briefly, every other day, at about eight o 'clock in the morning, freshly prepared 1 × 10^9^ CFU/mL *P. gingivalis* (0.2 mL/mice) were centrifuged and resuspended with 2% carboxymethylcellulose (CMC) solution. And then, the CMC-resuspended *P. gingivalis* were dipped and transferred onto the disposable oral-specific micro applicators. Finally, mice were orally infected with *P. gingivalis* by brushing the free gingival area slightly using the bacterial containing disposable oral-specific micro applicators. One week later, COPD models were started to construct using newly purchased cigarettes (Marlboro, 12 mg tar/1.0 mg nicotine; Philip Morris, Richmond, VA) and porcine pancreatic elastase (PPE, RHAWN catalog no. R028727). Mice were exposed to mainstream cigarette smoke (CS) for 2 h every day through the Cigarette Smoke Generators TSE system (TSE Systems China). Two days before CS exposure was begun and on the second day of CS exposure, mice under anesthesia underwent an intratracheal injection of phosphate-buffered saline (PBS) dissolved PPE (2.5 U/mouse each time, in total, 5 U/mouse). For the early-stage COPD model, mice were exposed to CS for 2 weeks, and for the late-stage COPD model, the CS exposure time was extended for an additional 2 weeks. For the anti-γδ T groups (6 mice/group for B group and P group; 9 mice/group for the other four COPD-associated groups), the mice were intraperitoneally injected with γδ-TCR monoclonal antibody (200 ug/mouse; BioXCell catalog no. BE0070) every 2–3 days.

### Lung function test

Lung function was measured with the EMMS eSpira Forced Maneuvers system according to the manufacture’s instruction. FEV0.05 value and FEV0.05/FVC value were used to compare the lung function of each group.

### H&E staining and methylene blue staining

Five-micrometer paraffin-embedded lung tissue sections were prepared for hematoxylin and eosin (H&E) staining with the H&E staining kit (Solarbio catalog no. G1120) to observe the morphological changes and to analyze disease severity.

The maxillae were stained with 1% methylene blue (Solarbio catalog no. G1303) as described by Wei et al. (35) and imaged with a microscope. The severity of bone resorption, represented by the distance from the cementoenamel junction (CEJ) to the alveolar bone crest (ABC), was measured to analyze periodontitis severity.

### 16S rRNA-sequencing

Freshly collected lung tissues were quickly frozen in liquid nitrogen and then transported on dry ice to the Personal Biotechnology company (Shanghai Personal Biotechnology Co., Ltd., China) for 16S RNA-sequencing (V3-V4) on the Illumina platform (Novaseq 6000). The data analysis was carried out with QIIME2 (2019.4).

### Tissue DNA extraction and *P. gingivalis* abundance detection

DNA extraction and *P. gingivalis* abundance detection was performed as described previously (74). Total DNAs of paraffin lung tissues were extracted with the QIAamp DNA Mini Kit (Qiagen, Dusseldorf, Germany). *P. gingivalis* abundances of lung tissues were measured through RT-qPCR (described later). The sequences of the *P. gingivalis* specific primers were Forward: 5′-AGGCAGCTTGCCATACTGCG-3′; Reverse: 5′-ACTGTTAGCAACTACCGATGT-3′.

### Immunofluorescence observation

Immunofluorescence staining and observation were performed as previously described (35). After xylene deparaffinization, gradient alcohol dehydration, hydrogen peroxide solution peroxidation, and goat serum block, the slides containing 5 μm-thick lung tissue slices were incubated with the following primary antibodies, second antibodies, and 49,6-diamidino-2-phenylindole (DAPI): anti-TCR γδ (BioLegend catalog no. 118101), anti-CD206 (Proteintech catalog no. 18704-1-AP), anti-CD86 (Bioss catalog no. bs-1035R), secondary antibodies (BioLegend catalog no. 405510; Proteintech catalog no. SA00013-2), and DAPI (Solarbio catalog no. C0065). Slides that completed the staining procedure were observed and imaged under an Olympus confocal microscope (FV31S-SW V2.4 software).

### Flow cytometry

The flow cytometry experiments were carried out according to the procedure outlined by Jin et al., with minor modifications, for analyzing γδ T activating and polarization of macrophage (29). Briefly, fresh single cell suspensions of lung tissues were firstly stained with the Fixable Viability kit (BioLegend catalog no. L423105 or eBioscience catalog no. 65-0866-18) and then blocked with anti-CD16/32 antibody (BioLegend catalog no. 101320). The cell suspensions were then incubated with the following flowcytometry antibodies (30–60 min): CD45 (BioLegend catalog no. 103139, Elabscience catalog no. E-AB-F1136Q), CD3 (BioLegend catalog no. 100204), F4/80 (BioLegend catalog no. 123130, Elabscience catalog no. E-AB-F0995Q, Elabscience catalog no. E-AB-F0995S), CD206 (BioLegend catalog no. 141706, Elabscience catalog no. E-AB-F1135E), CD86 (BioLegend catalog no. 105008), TCR γδ (BioLegend catalog no. 118118), IL 17 (BioLegend catalog no. 506916, Elabscience catalog no. E-AB-F1272Q), IFN γ (BioLegend catalog no. 113606, Elabscience catalog no. E-AB-F1101D). For intracellular cytokine staining (IL 17 and IFN γ), cells were pre-incubated with Cell Stimulation Cocktail (eBioscience, catalog no. 00-4970-93) for 6 h and Protein Transport Inhibitor Cocktail (eBioscience, catalog no. 00-4980-93) for 2 h (37°C, 5% CO_2_) before surface staining. After surface staining, fixation (BioLegend catalog no. 420801), and permeabilization (BioLegend catalog no. 421002), the cell suspension was intracellularly stained. Flow cytometry detection was performed on Attune NxT (Invitrogen Attune NxT flow cytometry software) and Cytoflex (Beckman Coulter Cytoflex flow cytometry software) flow cytometers, and data were analyzed by the FlowJo (V10.8) software and CytExpert software.

### Enzyme-linked immunosorbent assay (ELISA)

IL 17 (BioLegend catalog no. 436204) and IFN γ (BioLegend catalog no. 430804) ELISA kits were used to measure the levels of IL 17 and IFN γ in mouse serum samples, lung tissues samples, and the cell culture supernatant samples, respectively.

### RT-qPCR

For gene expression levels analysis, total RNA of each lung tissue sample was extracted with RNA extraction kit (Yeasen catalog no. 19221ES50) and reversely transcribed with RNA reverse kit (Yeasen catalog no. 11141ES60). Then, the cDNA samples were used for gene expression level analysis (*Gapdh* was used to normalize the expressions of different genes) with the RT-qPCR kit (Syber Green, Yeasen catalog no. 11201ES08). The expression levels were calculated with the 2^−△△*CT*^ method (75). The specific primers for the tested genes were designed using PrimerBanK (https://pga.mgh.harvard.edu/primerbank/) and synthesized by TSINGKE Biological Technology company (Sichuan, Chengdu), the sequences are listed in Table S3.

For the detection of *P. gingivalis* abundance in lung tissue, the gDNA obtained above underwent RT-qPCR procedure. *P. gingivalis* abundances were calculated with the 2^−△*CT*^ method (76) and displayed with fold changes to the blank control group.

### *Ex vivo* experiment

The lymphocytes of mouse lung tissues were extracted with the Percoll Cell separation fluid kit (Biosharp catalog no. BS909). BALF were collected with 1 mL sterile PBS and centrifuged to collect the cell precipitate. Lymphocytes or BALF cell precipitate were co-cultured with *P. gingivalis* in DMEM containing 10% FBS for 24 h.

The peripheral blood mononuclear cells (PBMC) of mice blood were extracted with Peripheral blood lymphocyte separation solution (Solarbio catalog no. P6340). PBMC were co-cultured with *P. gingivalis* for 24 h. For the anti-γδ T treatment *ex vivo* part, γδ-TCR monoclonal antibodies (200 ng/mL; BioXCell catalog no. BE0070) were added. Briefly, for the anti-γδ T treatment groups, the inhibitor was first intraperitoneally injected *in vivo*, and 24 h later, the peripheral blood of mice was collected to obtain the inhibitor-treated PBMC, and then, the obtained PBMC was cultured *in vitro*. For the corresponding control groups (without γδ-TCR monoclonal antibody treatment), an equal amount of PBS was intraperitoneally injected *in vivo*, 24 h later, the peripheral blood of mice was collected to obtain PBMC without inhibitor treatment, and then, the obtained PBMC was cultured *in vitro*.

Flow cytometry procedures were performed and analyzed as described above.

### Clinical sample analysis

The clinical samples were collected after the approval and supervision of the Medical Ethics Committee of West China Hospital of Stomatology, Sichuan University (WCHSIRB-D-2022-473). In total, 53 clinical BALF samples from COPD patients were collected at the Clinical Microbiology Lab of West China Hospital. After centrifugation, the precipitates and supernatants were collected and stored separately at −80℃ for future use. The precipitates were used for DNA extraction with the DNA extraction kit (Yeasen catalog no. 18700ES50) and RNA extraction with the same method and kit described above. The obtained gDNA was used for the RT-qPCR with the *P. gingivalis* specific primers as described above to assess whether the COPD BALF samples had a periodontitis associated state (CT > 35, indicates no detection of *P. gingivalis*, sample assigned to the COPD-no *P. g* group; CT < 35, indicates presence of *P. gingivalis*, sample assigned to the COPD-*P. g* group). The RNAs were used for gene expression analysis. The specific primers for the tested genes are listed in Table S4. The supernatants were collected for confirmation of IL 17 and IFN γ levels by ELISA with the anti-human IL 17 ELISA kit (MULTI SCIENCES catalog no. EK117HS-96) and IFN γ ELISA kit (MULTI SCIENCES catalog no. EK180HS-96), respectively.

### Statistical analysis

Data in this study were represented as the mean ± standard deviation (SD) for at least three independent samples in each group. For the second batch of animal experiments (only two groups: C group and CP group), the *ex vivo* experiments (except the anti-γδ T treatment portion) and the clinical sample measurement, after a homogeneity test of variance with Levene’s test, *t* test, or Kruskal-Wallis analysis were used for analysis of differences. For the other experiments, including the multiple-group animal models and four-group *ex vivo* experiments, one-way ANOVA, and post hoc Tukey’s multiple comparisons were used for analysis of differences among multiple groups, and *t* test was used for two independent groups. Statistical analysis was performed using GraphPad Prism7 software (version 7.00 for Windows; GraphPad Prism, Inc, La Jolla, CA, USA) with a significance level of 0.05, and all figures were also generated with this software.

## Data Availability

The 16S rRNA sequencing data have been deposited in the public database Sequence Read Archive with accession no. PRJNA925666. All data generated or analyzed in this study are available from the corresponding author on reasonable request.

## SUPPLEMENTAL MATERIAL

Supplemental Tables and Figures msystems.00572-23-s0001.docx Tables S1 to S4; Fig. S1 to S8.

ASM does not own the copyrights to Supplemental Material that may be linked to, or accessed through, an article. The authors have granted ASM a non-exclusive, world-wide license to publish the Supplemental Material files. Please contact the corresponding author directly for reuse.
