# Supplementary material for: Periodontitis aggravates COPD through the activation of γδ T cell and M2 macrophage
Source: mSystems. 2024 Jan 12;9(2):e00572-23. doi: 10.1128/msystems.00572-23 (PMC10878042; doi:10.1128/msystems.00572-23)
Supplement: Supplemental Tables and Figures — Tables S1 to S4; Fig. S1 to S8. [file msystems.00572-23-s0001.docx]

**Periodontitis aggravates COPD through the activation of γδ T cell and M2 macrophage**

**Authors:** Kaixin Xiong^1, #^, Keping Ao ^2, #^, Wei Wei^1,3^, Jiajia Dong^4^, Jia Li^1^, Yutao Yang^1^, Boyu Tang^1, 5,^ *, Yan Li^1,^ *

^1^ State Key Laboratory of Oral Diseases & National Center for Stomatology & National Clinical Center for Oral Diseases, West China Hospital of Stomatology, Sichuan University, Chengdu, 610041, China

^2^ Department of Laboratory Medicine, West China Hospital, Sichuan University, Chengdu, China.

^3^ Department of Prosthodontics, Beijing Stomatological Hospital, School of Stomatology, Capital Medical University, Beijing, China.

^4^ Department of Pulmonary and Critical Care Medicine, West China Hospital, Sichuan University, Chengdu, China.

^5^ Department of Conservation Dentistry and Endodontics, West China Hospital of Stomatology, Sichuan University, Chengdu, 610041, China

^#^ These authors contributed equally to this work.

***Corresponding Author:**

Dr. Yan Li, State Key Laboratory of Oral Diseases & National Center for Stomatology & National Clinical Center for Oral Diseases, West China Hospital of Stomatology, Sichuan University, Chengdu, 610041, China (Email: [feifeiliyan@163.com](mailto:feifeiliyan@163.com)).

Dr. Boyu Tang, State Key Laboratory of Oral Diseases & National Center for Stomatology & National Clinical Center for Oral Diseases & Department of Conservation Dentistry and Endodontics, West China Hospital of Stomatology, Sichuan University, Chengdu, 610041, China (Email: [boyutang@126.com](mailto:boyutang@126.com)).

**Supplementary Table S1: Relative abundance of several periodontal inflammation associated bacteria in the lung tissue through 16S rRNA-Sequencing analysis**

| Relative abundance (%) | COPD | COPD with periodontitis |
| --- | --- | --- |
| Bacteroidetes | 2.063 ± 1.698 | 2.917 ± 1.543 |
| Bacteroides | 0.2933 ± 0.2183 | 0.5200 ± 0.8920 |
| Prevotella | 0.1400 ± 0.1997 | 0.2167 ± 0.1930 |

**Supplementary Table S2: Characterization of the clinical subjects in the two groups in this study**

|  | COPD-no P.g group | COPD-P.g group |
| --- | --- | --- |
| Number of samples | 28 | 25 |
| Age (year) | 70.18±10.28 years old | 69.92±11.9 years old |
| Sex | females: 2 samples  males: 26 samples | female: 1 sample  males: 24 samples |
| Smoke history | Yes: 20 samples  No: 8 samples  Mean: 34.5±13.23 years | Yes: 19 samples  No: 6 samples  Mean: 34.74±13.38 years |
| Hypertension | 4 samples | 5 samples |
| Diabetes | 3 samples | 3 samples |
| Heart disease | 5 samples | 7 samples |
| Infectious diseases | None | None |
| Lung Tumors | None | None |
| COPD Acute exacerbation | Yes: 19 samples  No: 8 samples | Yes: 20 samples  No: 5 samples |
| Acute exacerbation rate (%) | 67.86% | 80.00% |

**Supplementary Table S3. RT-qPCR primers used for gene expressions in mice in this study**

| Genes | Primers | Sequences (5’–3’) |
| --- | --- | --- |
| *Gapdh* | *Gapdh*-F  *Gapdh*-R | AGGTTGTCTCCTGCGACTTCA  CCAGGAAATGAGCTTGACAAA |
| *IL 17* | *IL 17*-F  *IL 17*-R | CAGGACGCGCAAACATGA  GCAACAGCATCAGAGACACAGAT |
| *Ifn γ* | *Ifn γ*-F  *Ifn γ*-R | ATGAACGCTACACACTGCATC  CCATCCTTTTGCCAGTTCCTC |
| *CD86* | *CD86*-F  *CD86*-R | TGTTTCCGTGGAGACGCAAG  TTGAGCCTTTGTAAATGGGCA |
| *iNOS* | *iNOS* -F  *iNOS* -R | GTTCTCAGCCCAACAATACAAGA  GTGGACGGGTCGATGTCAC |
| *IL 1β* | *IL 1β*-F  *IL 1β*-R | CAACCAACAAGTGATATTCTCCATG  ATCCACACTCTCCAGCTGCA |
| *IL 23* | *IL 23*-F  *IL 23*-R | ATGCTGGATTGCAGAGCAGTA  ACGGGGCACATTATTTTTAGTCT |
| *CD206* | *CD206*-F  *CD206*-R | CTCTGTTCAGCTATTGGACGC  CGGAATTTCTGGGATTCAGCTTC |
| *Arg 1* | *Arg 1*-F  *Arg 1*-R | CTCCAAGCCAAAGTCCTTAGAG  AGGAGCTGTCATTAGGGACATC |
| *Mmp 9* | *Mmp 9*-F  *Mmp 9*-R | CTGGACAGCCAGACACTAAAG  CTCGCGGCAAGTCTTCAGAG |
| *Mmp 12* | *Mmp 12*-F  *Mmp 12*-R | CTGCTCCCATGAATGACAGTG  AGTTGCTTCTAGCCCAAAGAAC |
| *IL 4* | *IL 4*-F  *IL 4*-R | GGTCTCAACCCCCAGCTAGT  GCCGATGATCTCTCTCAAGTGAT |

**Supplementary Table S4. RT-qPCR primers used for gene expressions in human samples in this study**

| Genes | Primers | Sequences (5’–3’) |
| --- | --- | --- |
| *GAPDH* | *GAPDH*-F  *GAPDH*-R | ACAACTTTGGTATCGTGGAAGG  GCCATCACGCCACAGTTTC |
| *IL 17* | *IL 17*-F  *IL 17*-R | AGATTACTACAACCGATCCACCT  GGGGACAGAGTTCATGTGGTA |
| *IFN γ* | *IFN γ*-F  *IFN γ*-R | TCGGTAACTGACTTGAATGTCCA  TCGCTTCCCTGTTTTAGCTGC |
| *CD206* | *CD206*-F  *CD206*-R | GGGTTGCTATCACTCTCTATGC  TTTCTTGTCTGTTGCCGTAGTT |
| *Arg 1* | *Arg 1*-F  *Arg 1*-R | TGGACAGACTAGGAATTGGCA  CCAGTCCGTCAACATCAAAACT |
| *Mmp 9* | *Mmp 9*-F  *Mmp 9*-R | TGTACCGCTATGGTTACACTCG  GGCAGGGACAGTTGCTTCT |
| *Mmp 12* | *Mmp 12*-F  *Mmp 12*-R | GGAATCCTAGCCCATGCTTTT  CATTACGGCCTTTGGATCACT |
| *IL 4* | *IL 4*-F  *IL 4*-R | CCAACTGCTTCCCCCTCTG  TCTGTTACGGTCAACTCGGTG |
| *IL 10* | *IL 10*-F  *IL 10*-R | GACTTTAAGGGTTACCTGGGTTG  TCACATGCGCCTTGATGTCTG |
| *IL 13* | *IL 13*-F  *IL 13*-R | GAAGGCTCCGCTCTGCAAT  TCCAGGGCTGCACAGTACA |
| *TGF β* | *TGF β*-F  *TGF β*-R | GGCCAGATCCTGTCCAAGC  GTGGGTTTCCACCATTAGCAC |

| 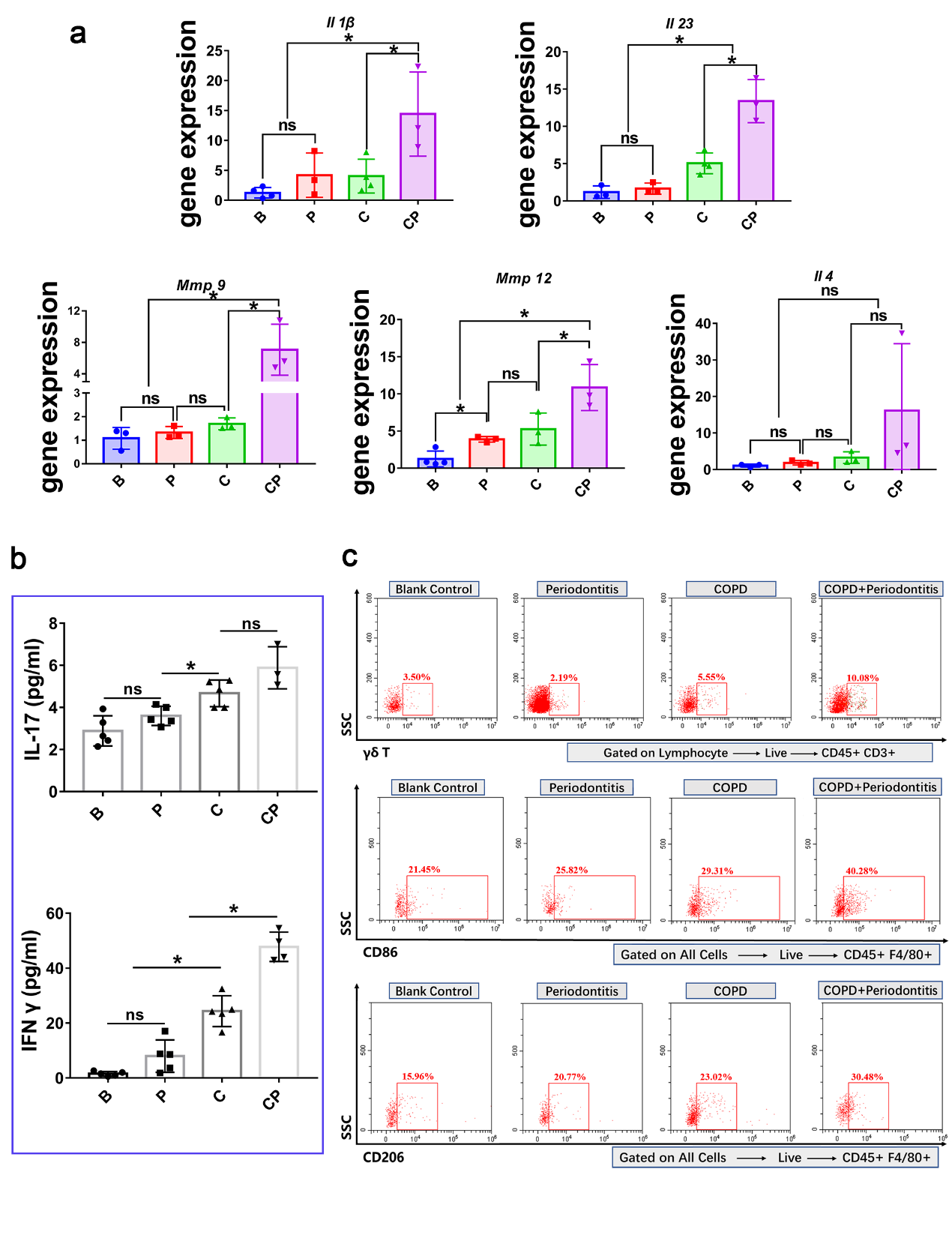 |
| --- |

**Supplementary Figure S1: Figure 3-related supplementary figure.**

(a): In the early stage of COPD, periodontitis promoted the expression of M1 and M2 associated inflammatory cytokines in COPD lung tissue. (b): In the early stage of COPD, IL 17 and IFN γ levels in serum of each group were calculated by ELISA. (c): In the early stage of COPD, percentages of γδ T positive cells, M1 polarized macrophages and M2 polarized macrophages in the lung tissue of each group were analyzed by flow cytometry and representative flow cytometry plots were shown. *: P < 0.05, ns: not significant. B: Blank Control, P: periodontitis, C: COPD, CP: COPD with periodontitis.

| 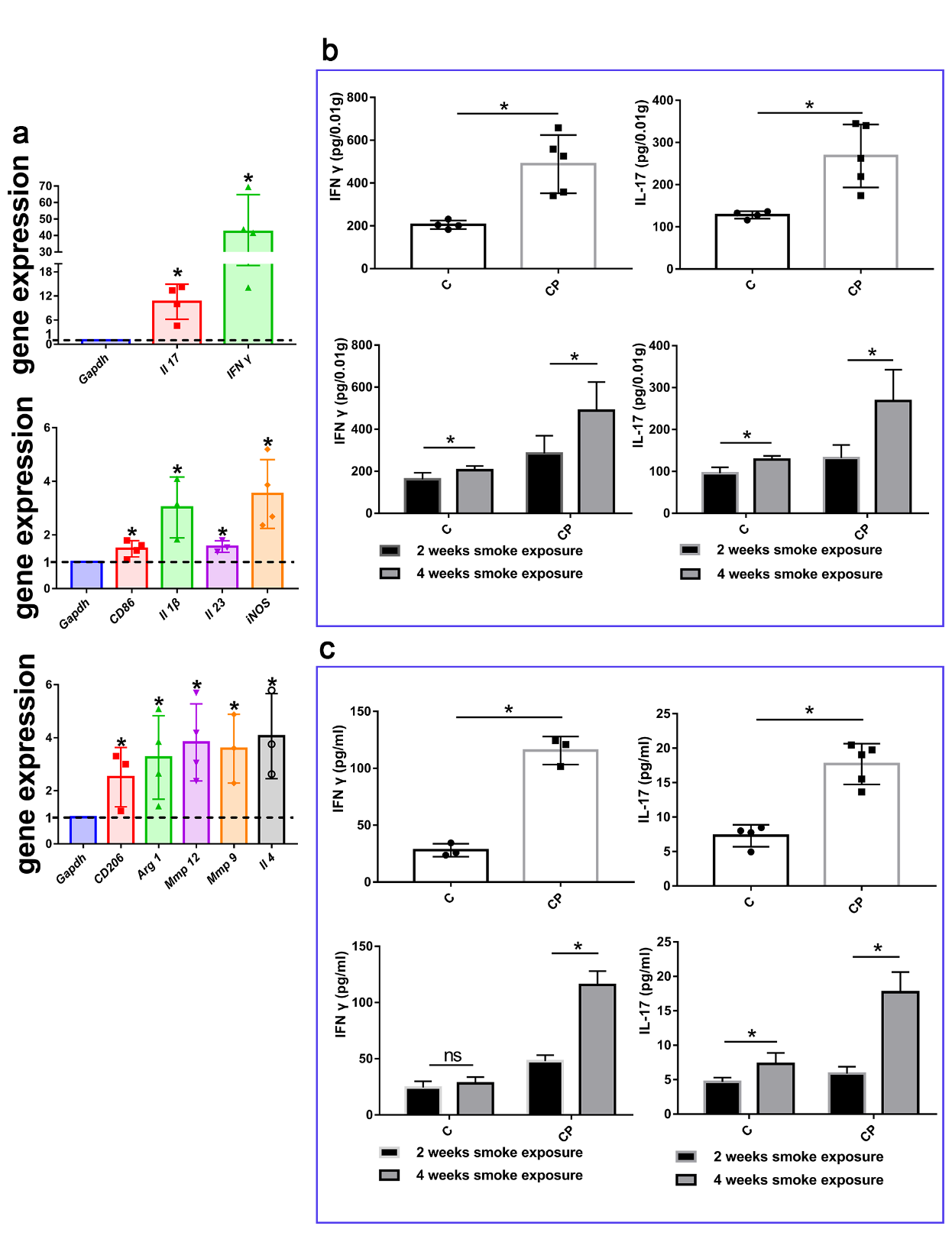 |
| --- |

**Supplementary Figure S2: Figure 3-related supplementary figure. Gene expression levels, as well as the levels of IL 17 and IFN γ were further upregulated with extended (4 weeks) smoke exposure.** (a): Gene expression level analysis of the lung tissue under the extended smoke exposure state. (b, c): Under the extended smoke exposure state, the levels of IFN γ and IL 17 in lung tissue (b) and serum (c) were determined by ELISA and compared with the corresponding short time smoke exposure (2 weeks) samples. *: P < 0.05, ns: not significant. C: COPD, CP: COPD with periodontitis.

| 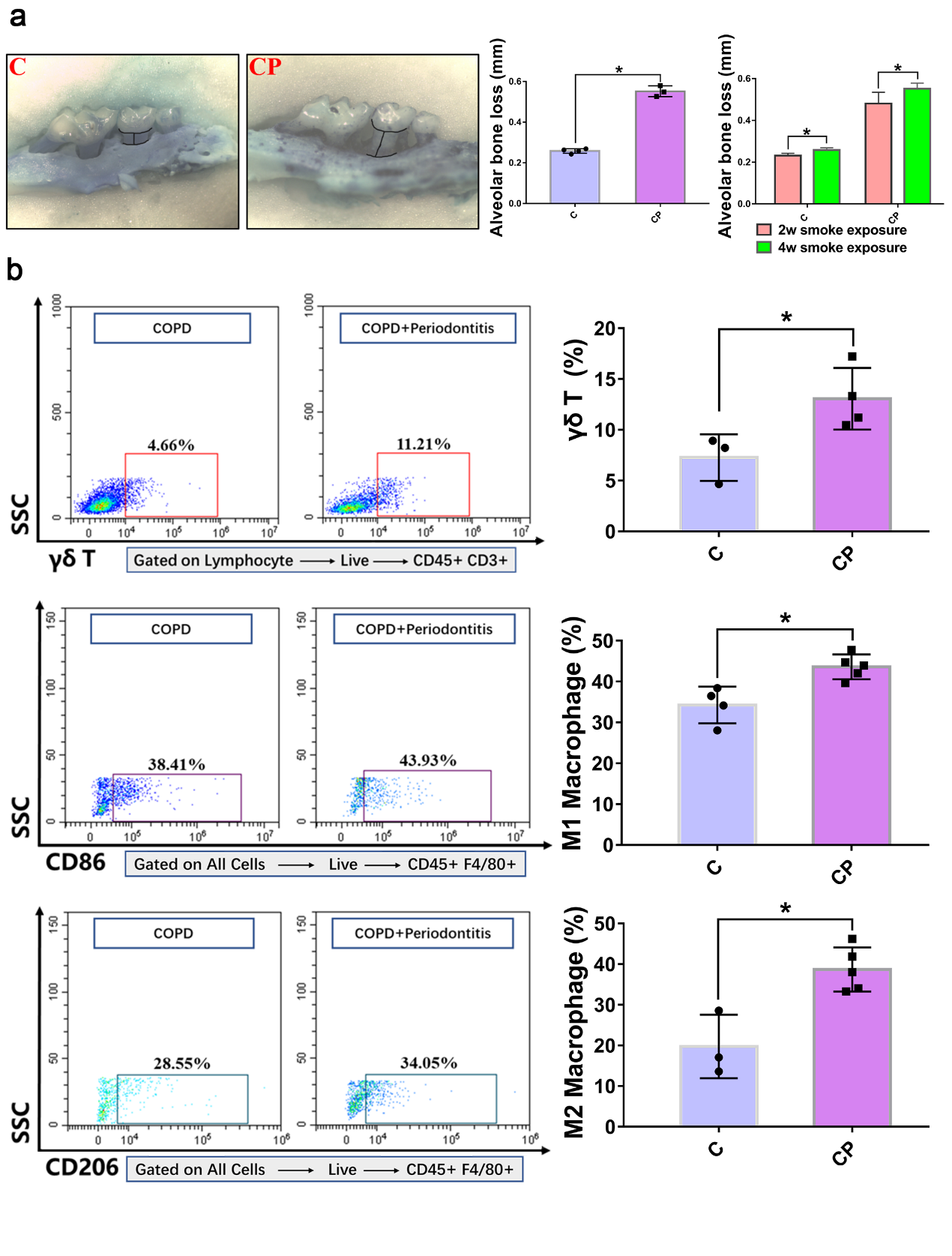 |
| --- |

**Supplementary Figure S3: Figure 3-related supplementary figure.**

(a): In the late-stage COPD model, the alveolar bone loss was further increased. Representative images and the quantification and comparison with the corresponding results of the early-stage COPD model were shown. (b): In the late stage of COPD, flow cytometry analysis was carried out to analyze γδ T positive cells, M1 polarized macrophages and M2 polarized macrophages in the lung tissue of each group, and representative flow cytometry plots and the corresponding quantitative analyses were shown. *: P < 0.05, ns: not significant. C: COPD, CP: COPD with periodontitis.

| 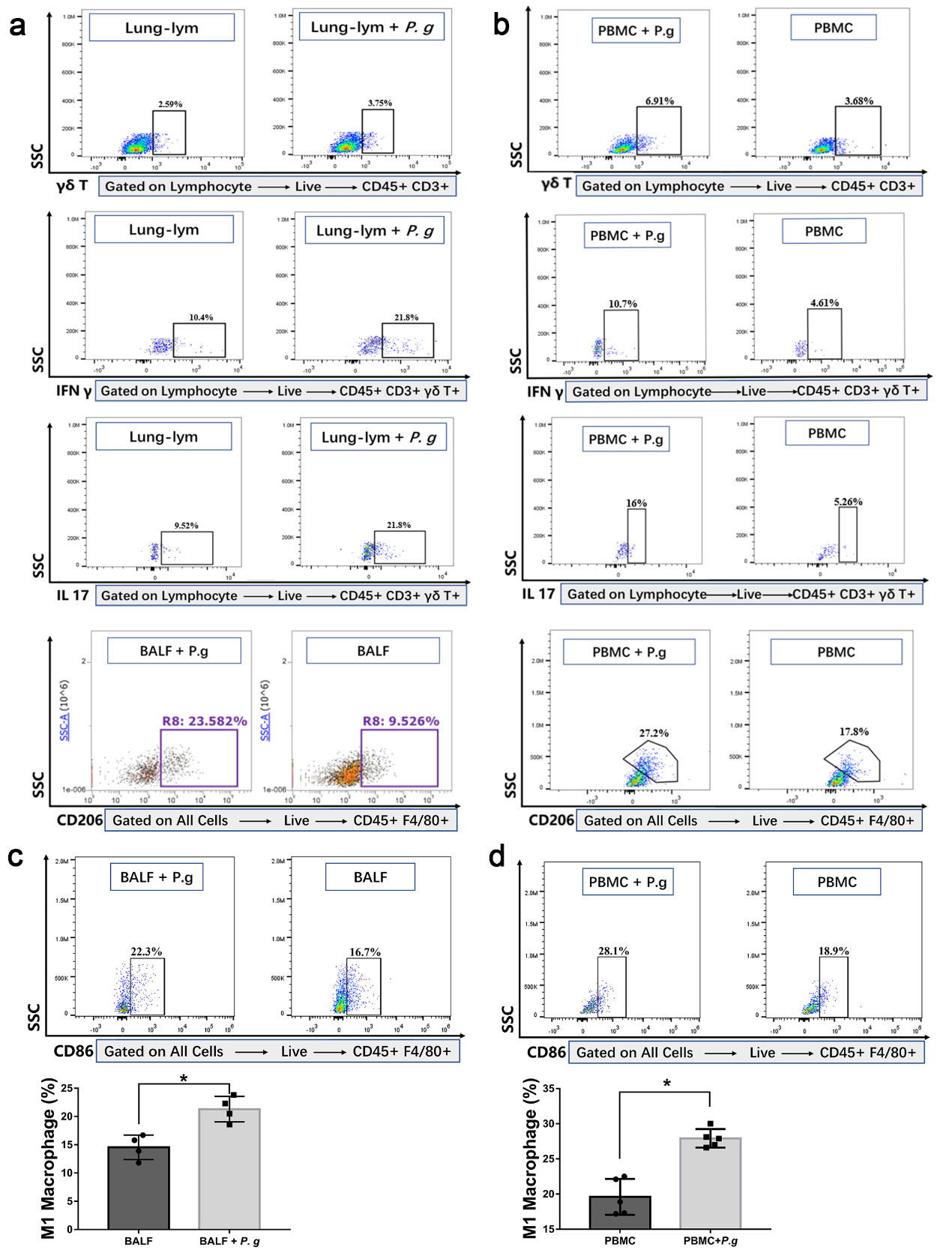 |
| --- |

**Supplementary Figure S4: Figure 4-related supplementary figure.**

(a, c): Flow cytometry analysis indicated that *P. gingivalis* promoted γδ T cells activation in the lymphocytes of lung tissue, and M1 macrophages and M2 macrophages in the BALF. Representative flow cytometry plots were shown. (b, d): Flow cytometry analysis indicated that *P. gingivalis* promoted γδ T cells activation, and M1 macrophages and M2 macrophages in the PBMCs. Representative flow cytometry plots were shown. Lung-lym: the lymphocytes of lung tissue, Lung-lym + *P. g*: the lymphocytes of lung tissue cocultured with *P. gingivalis,* BALF: Bronchoalveolar lavage fluid, BALF + *P. g*: Bronchoalveolar lavage fluid cocultured with *P. gingivalis*, PBMC: Peripheral blood mononuclear cell, PBMC + *P.g*: Peripheral blood mononuclear cell cocultured with *P. gingivalis.*

| 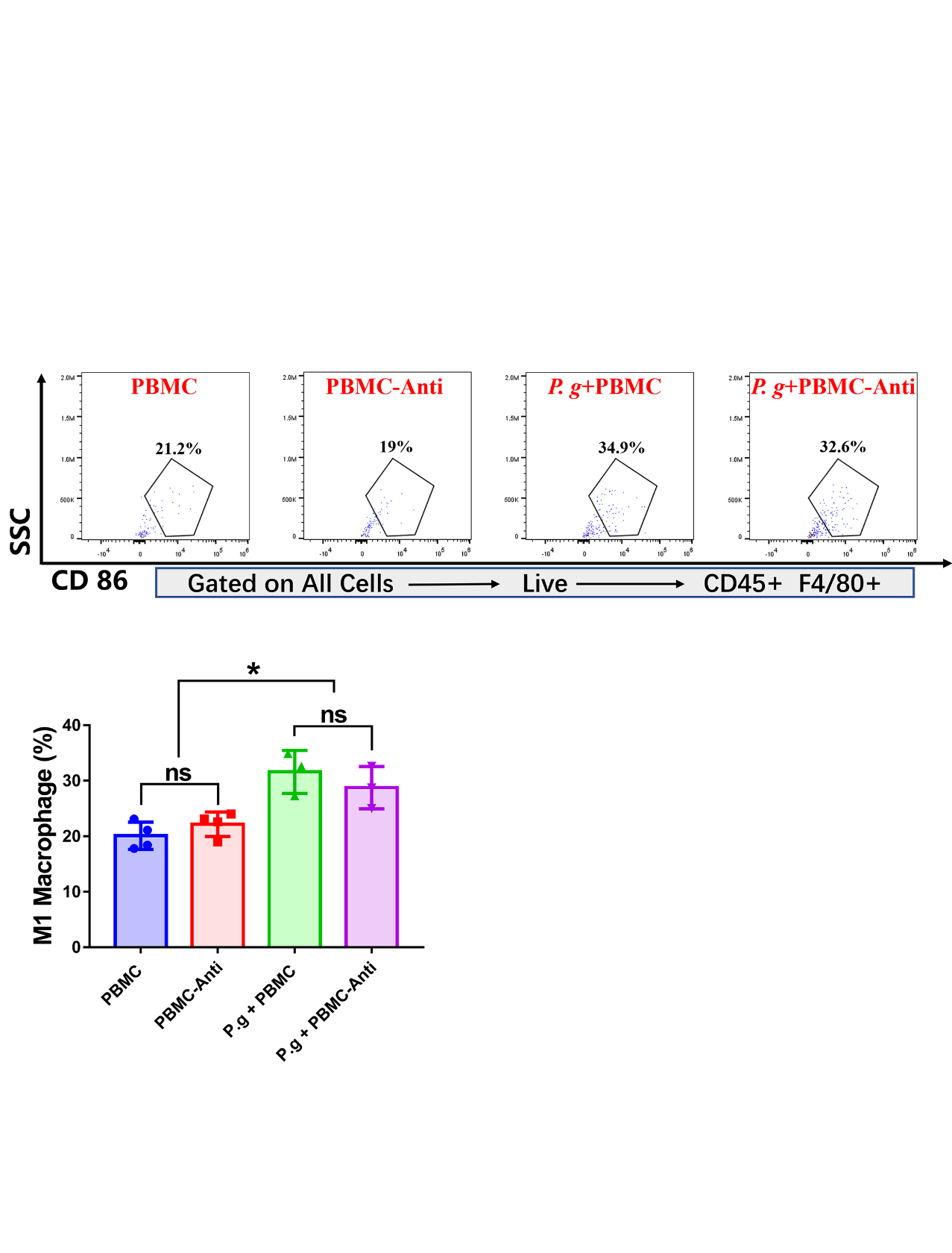 |
| --- |

**Supplementary Figure S5: Figure 4-related supplementary figure.**

With or without γδ-TCR monoclonal antibody treatment, *P. gingivalis* were cocultured with PBMCs. The M1 polarization was analyzed by flow cytometry and representative flow cytometry plots were shown, and the quantitative analysis of M1 cells were presented as Mean ± STD. *: P < 0.05, ns: not significant. PBMC: Peripheral blood mononuclear cell, PBMC + *P. g*: Peripheral blood mononuclear cell cocultured with *P. gingivalis*, PBMC-Anti: PBMC group with γδ-TCR monoclonal antibody treatment, *P. g* + PBMC-Anti: *P. g* + PBMC group with γδ-TCR monoclonal antibody treatment.


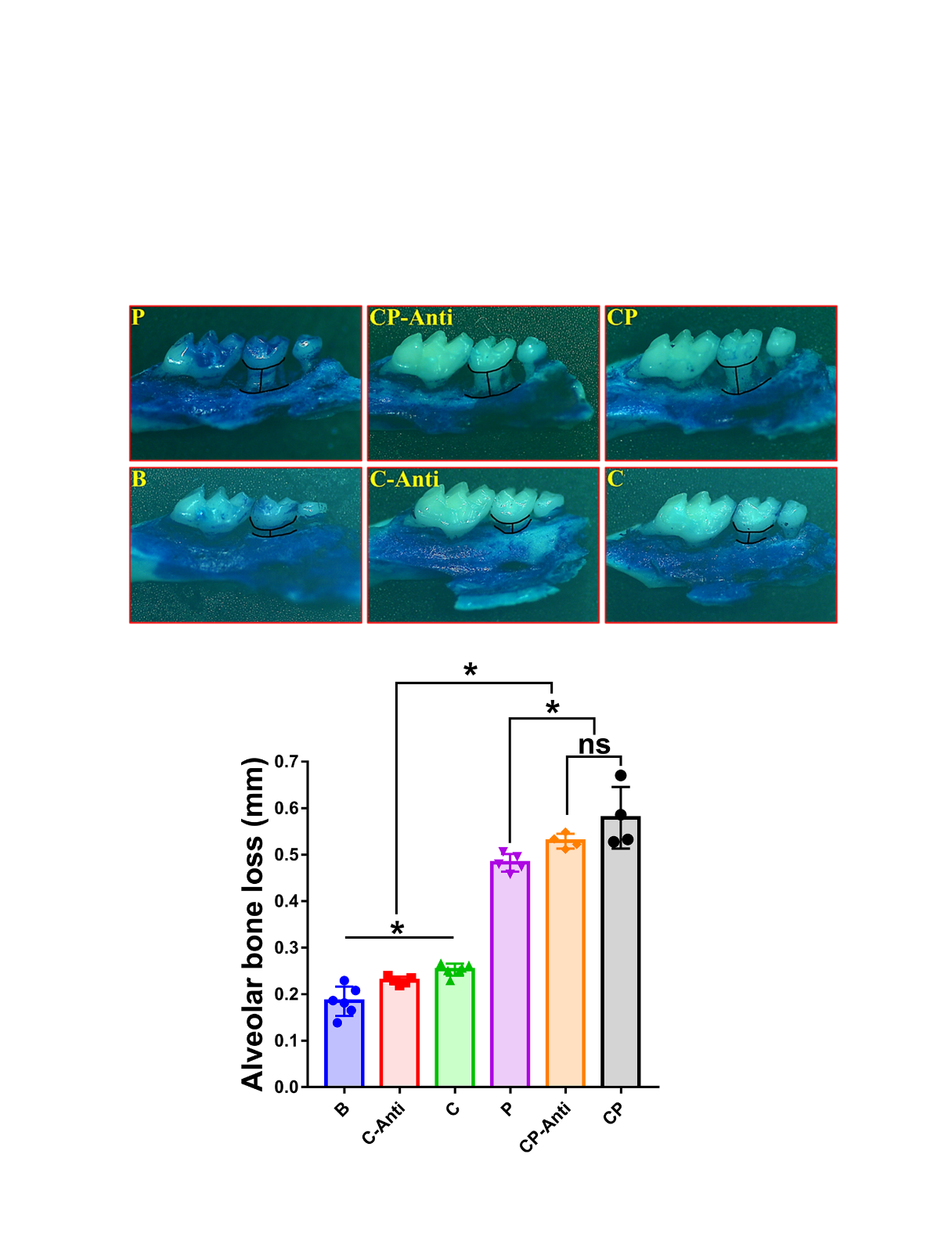


**Supplementary Figure S6: Figure 5-related supplementary figure.**

With or without γδ-TCR monoclonal antibody treatment, the methylene blue stain was carried out to analyze the periodontitis alveolar bone loss and a representative image of the jaw for each group and quantitative analysis of alveolar bone loss were shown. *: P < 0.05, ns: not significant. B: Blank Control, P: periodontitis, C: COPD, CP: COPD with periodontitis, C-Anti: the COPD group with γδ-TCR monoclonal antibody treatment, CP-Anti: the CP group with γδ-TCR monoclonal antibody treatment.


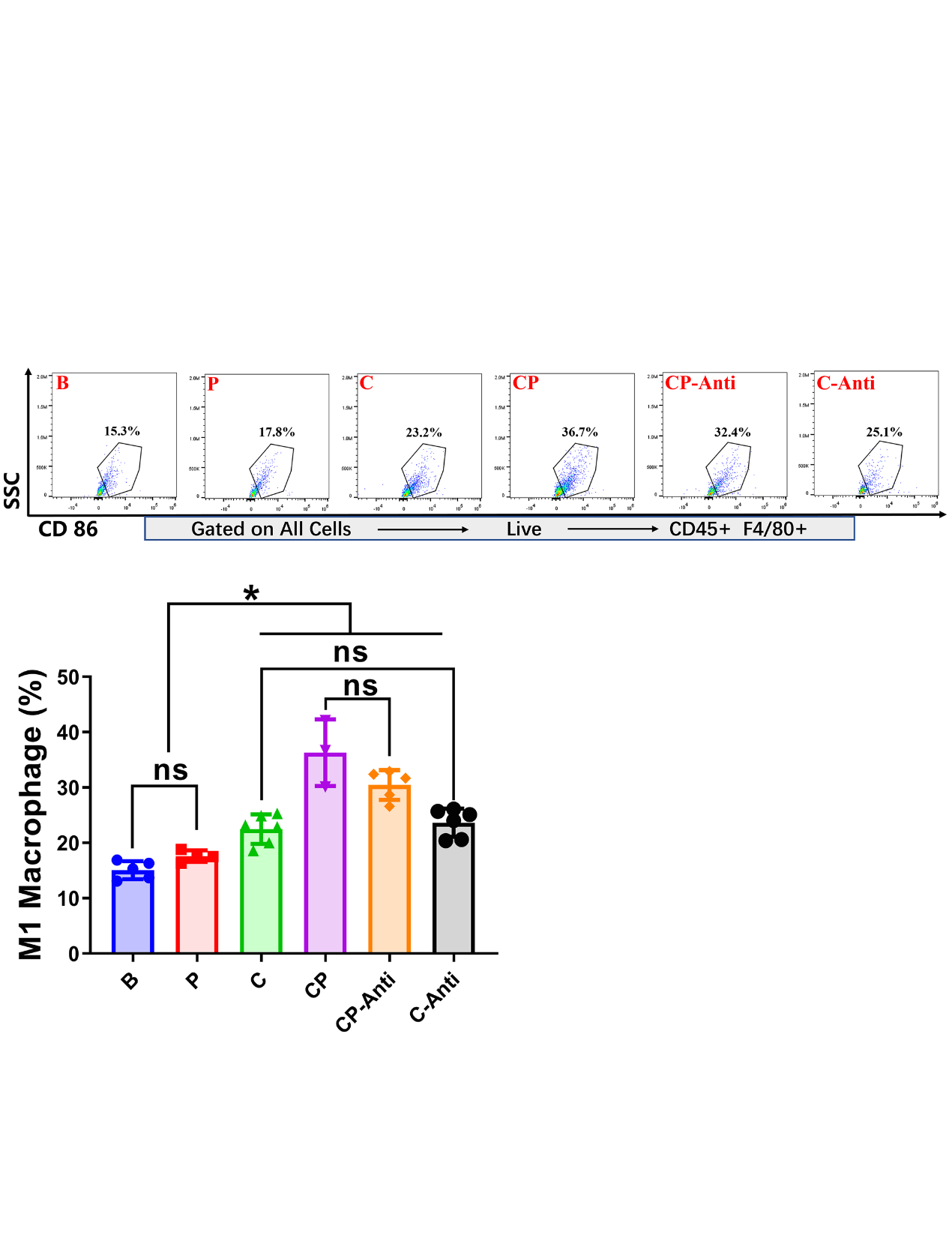


**Supplementary Figure S7: Figure 5-related supplementary figure.**

With or without γδ-TCR monoclonal antibody treatment, representative flow cytometry plots and the corresponding quantitative analysis of lung tissue in each group were shown. *: P < 0.05, ns: not significant. B: Blank Control, P: periodontitis, C: COPD, CP: COPD with periodontitis, C-Anti: the COPD group with γδ-TCR monoclonal antibody treatment, CP-Anti: the CP group with γδ-TCR monoclonal antibody treatment.

| 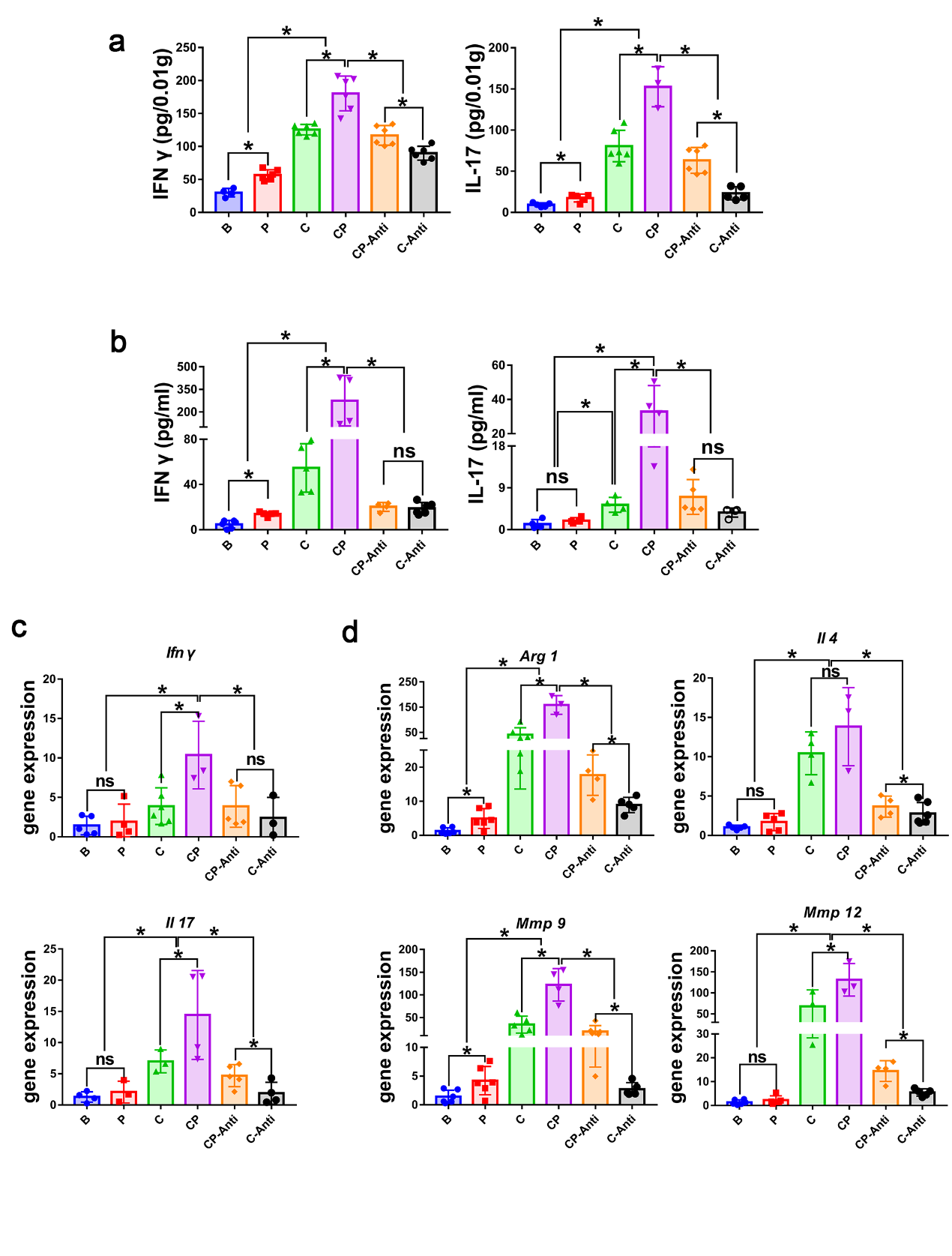 |
| --- |

**Supplementary Figure S8: Figure 5-related supplementary figure. γδ-TCR monoclonal antibody treatment decreased IL 17 and IFN γ levels and gene expression levels *in vivo*.**

(a, b): IL 17 and IFN γ levels in lung tissue and serum were measured by ELISA. (c, d): Relative expression levels of γδ T-related genes (c) and M2-related genes (d) in the lung tissue were quantified by RT-qPCR. *: P < 0.05, ns: not significant. B: Blank Control, P: periodontitis, C: COPD, CP: COPD with periodontitis, C-Anti: the COPD group with γδ-TCR monoclonal antibody treatment, CP-Anti: the CP group with γδ-TCR monoclonal antibody treatment.
